# Antipsychotics for Amphetamine Psychosis. A Systematic Review

**DOI:** 10.3389/fpsyt.2019.00740

**Published:** 2019-10-15

**Authors:** Dimy Fluyau, Paroma Mitra, Kervens Lorthe

**Affiliations:** ^1^School of Medicine, Emory University, Atlanta, GA, United States; ^2^Langone Health, Department of Psychiatry, NYU, New York, NY, United States; ^3^Department of Health, Miami Regional University, Miami Springs, FL, United States

**Keywords:** amphetamine psychosis, psychosis, addictive disorders, treatment outcome, risk, amphetamine, antipsychotics

## Abstract

**Background:** Among individuals experiencing amphetamine psychosis, it may be difficult to rule out schizophrenia. The use of antipsychotics for the treatment of amphetamine psychosis is sparse due to possible side effects. Some arguments disfavor their use, stating that the psychotic episode is self-limited. Without treatment, some individuals may not fully recover from the psychosis and may develop full-blown psychosis, emotional, and cognitive disturbance. This review aims to investigate the clinical benefits and risks of antipsychotics for the treatment of amphetamine psychosis.

**Methods:** Electronic search on trials on antipsychotic drugs for amphetamine psychosis from their inception to November 2018 was conducted in PubMed, Scopus, Google Scholar, EBSCOhost, ProQuest, Cochrane Review Database, Medline Ovid, and EMBASE following the Preferred Reporting Items for Systematic Reviews and Meta-Analysis guidelines. The Cochrane risk-of-bias tool assessed the risk of bias, the methodological quality of individual trials was assessed by the Oxford Quality Scoring System, and the quality of evidence for recommendations was judged by the Grading of Recommendations, Assessment, Development, and Evaluations (GRADE). The results were synthesized qualitatively and quantitatively.

**Results:** The investigation of six randomized controlled trials of 314 participants showed that aripiprazole, haloperidol, quetiapine, olanzapine, and risperidone were able to reduce or control the psychotic episode (positive and negative symptoms) induced by amphetamine use with no adverse event. Although the side-effect profile of these agents varied, no drug was clinically superior to others.

**Conclusions:** This review suggests that antipsychotics seem to be efficacious for amphetamine psychosis on both positive and negative symptoms. Practitioners need to tailor their use based on risks for side effects individually.

## Introduction

**Rationale:** The use of amphetamine encompasses several street drugs that fall under the umbrella of crystal meth, crank, speed, tweek, glass, and so forth, and those with a substituted-phenylethylamine structure, such as amphetamine, dextroamphetamine, and methamphetamine. It also involves the use of other different structures like methylphenidate for the treatment of attention deficit hyperactivity disorder and narcolepsy and designer drugs with amphetamine-type compounds like bath salts, molly, and flakka. Amphetamine use also implicates several plant-derived stimulants with amphetamine’s chemical structure found in khât and kratom. Fenethylline, ephedrine, pseudoephedrine, and 3,4-methylenedioxymethamphetamine or ecstasy are also in the amphetamine category.

A letter to the editor published in 1957 drew attention to the widespread abuse of amphetamine in Great Britain and cases of amphetamine intoxication that could induce paranoia indistinguishable from the symptom of paranoia in schizophrenia (1). According to the United Nations Office on Drugs and Crime (2), East and South-East Asia and North America are the central subregions for methamphetamine trafficking worldwide. In 2016, methamphetamine was second after heroin as a drug threat in the United States of America. Worldwide, amphetamine and prescription stimulants reached 34 million among past-year users in 2016, and around 4 out of 10 methamphetamine users experienced psychosis (3). Among the group of users experiencing amphetamine-induced psychotic disorder (amphetamine psychosis), it may be difficult to rule out schizophrenia. The resolution of the psychotic episode may be incomplete without treatment. The risk of relapse is elevated (4–6). There are reports that methamphetamine users are more susceptible to exhibiting psychotic symptoms than the general population (7), and the psychosis may persist up to 6 months even after abstinence (8). It is not sure whether amphetamine psychosis is categorically different from other primary psychoses like schizophrenia, though it seems there is a similarity between the two conditions (9).

Evidence that stimulants may produce long-term psychosis was better supported in animals than in humans (10). Arunogiri et al. (11) reported that there is moderate evidence that the frequency of use of methamphetamine and the severity of dependence to the drug can increase the chance of developing amphetamine psychosis. The use of amphetamine itself may increase the risk of death due to intoxications, accidents, suicide and homicide, and cardiovascular events such as prolonged QTc interval (12–17). Amphetamine psychosis is prevalent among individuals who use the drug. A meta-analysis estimated that 36.5% of methamphetamine users have a history of amphetamine psychosis or substance-induced psychotic disorders (18).

### Psychopathology of Amphetamine Psychosis

The cascade of neurobiological events leading to the psychosis after amphetamine exposure is complicated. Among several molecules involved in amphetamine psychosis, dopamine (DA) seems to be primordial to the development of the psychotic phenomenon *via* the nigrostriatal DA system. However, serotonin, norepinephrine, opiate peptide–DA interactions, and amino acids are other neurotransmitter systems implicated in the biological basis of amphetamine psychosis (19). Amphetamine causes an excessive release of DA, leading to glutamate overflow. The overflow of glutamate damages the gamma-aminobutyric acid (GABA) interneurons; subsequently, the loss of GABA neurons causes glutamate dysregulation in the cortex, thus the development of psychosis (20). The cortical interneurons have a high proportion of extrasynaptically modulated N-methyl-D-aspartate (NMDA) receptors. These NMDA receptors are more vulnerable to neurotoxicity. Amphetamine or MDMA has been shown to be neurotoxic to domapine2 (D2) receptor in knock-out mice (21). It is suggested that the neurotoxicity induced by MDMA can cause untreatable chronic psychosis in human users (22). Continuous use of amphetamine can deplete the nigrostriatal DA system, resulting in a reduction in striatal tyrosine hydroxylase (TH) activity and a fall in the number of striatal DA receptors (19, 23–27). Bioimaging and histopathologic evaluations not only correlated clinical findings with damage to DA but also correlated clinical findings to damage to serotonin axons (presence of hypertrophy of the white matter and microgliosis in different brain areas) (28). Amphetamine treatment (3 mg/kg/day for 30–50 days) increased serotonin levels in cats (28, 29). Positron emission tomography (PET) studies showed a reduction of DA transporter density, serotonergic transporter density, and vesicular monoamine transporter (30). Overall, amphetamine psychosis is a complex phenomenon affecting the catecholaminergic systems, especially: DA, 5-hydroxytryptamine, and norepinephrine.

Genetic variations may also explain the complexity of amphetamine psychosis. Grant et al. (28) reported a group of genes strongly associated with amphetamine psychosis: d-amino acid oxidase activator, dystrobrevin-binding protein 1, frizzled 3, metabotropic glutamate receptor 2, 5-hydroxytryptamine (serotonin) receptor 1A, prokineticin receptor 2, and glycine transporter 1. Among these genes, four involve glutamatergic neurotransmission D-amino acid oxidase activator (DAOA), Dystrobrevin-binding protein 1 (DTNBP1), Metabotropic glutamate receptor 2 (GRM2) and Solute carrier family 6 member 9 (SLC6A9).

Amphetamine psychosis is a complex neurobiological event demonstrated in several experiments in animals and humans. Amphetamine psychosis can cause permanent damage to the brain (31, 32).

### Symptoms of Amphetamine Psychosis

**Table 1** depicts a group of symptoms of amphetamine psychosis. A salient feature of amphetamine psychosis is that the clinical manifestation is apparent during the time an individual is under the influence of the drug, but the clinical manifestation disappears when the drug is no longer in the body. During the syndromal episode, the psychosis is mostly indistinguishable from schizophrenia (36). Bell (33) reviewed 15 cases of individuals exposed to amphetamine and noted that visual hallucinations were prominent. There are anecdotal reports of individuals seeing giant snakes that are biting them and demons from hell ripping off their soul over and over. Amphetamine users experience a high frequency of persecutory delusions, delusions of jealousy, delusions of reference, delusions of mind-reading, agitation, visual and auditory hallucinations, thought insertion and thought broadcasting, derealization, and depersonalization (5, 6, 33, 34, 37). In 152 participants diagnosed with methamphetamine-induced psychosis, delusions of persecution (85.5%), violence (75.6%), intimate partner violence (61.2%), and auditory hallucinations (51.3%) were prominent (34). Users of amphetamine may be at higher risk for injury during the psychotic episode. During the psychosis, users can also become a safety risk for others.

**Table 1:** Depicts the symptoms most frequently encountered in amphetamine psychosis.

Symptoms of amphetamine psychosis			
Symptoms			Amphetamine psychosis
			(frequency)
Positive symptoms			
Auditory hallucination			++++
Delusions of persecution			++++
Disorganized thinking			+++
Psychomotor agitation			++++
Hostility			++
Grandiosity			++
Delusions of influence			+
Euphoria			++
Exhibitionism			+
Delusions of reference			++++
Visual hallucinations			++++
Delusions of jealousy			+
Compulsive thoughts			+
Negative symptoms			
Poverty of speech			+
Psychomotor retardation			+
Depression			+
			+
Others			
Derealization/depersonalization			+
Anxiety (disorders)			+

++++, very frequently; +++, frequently; ++, occasionally; +, rarely.

Reference # 5: Exploratory factor analysis (Manchester scale). Factor 1: table 2, page 961. (5)

Reference # 6: Positive and Negative Syndrome Scale (PANSS). Mean score. Table 2, page 20. (6)

Reference # 33: Description of 14 cases of amphetamine psychosis. Page 703. (33)

Reference # 34: Clinical symptom profiles of patients with methamphetamine psychosis. Table 2, page 1411. (34)

Reference # 35: Clinical features, course, and treatment of methamphetamine-induced psychosis in psychiatric inpatients. Table 2, page 4. (35)

### What Are All the Current Treatments of Amphetamine Psychosis Other Than Antipsychotics?

There is no established guideline for the treatment of amphetamine psychosis. In an acute setting, supportive management and pharmacological interventions are both recommended for individuals with amphetamine psychosis. The primary treatment goal is abstinence from amphetamine (10, 38). A benzodiazepine agonist or a histamine agonist is commonly used for excessive agitation. Interestingly, electroconvulsive therapy (ECT) has been suggested to be useful. In a case study of a 24-year-old man, the psychotic symptoms induced by methamphetamine completely disappeared after four sessions of ECT (39). In another case study of a 37-year-old man, improvement with ECT was seen after four right unilateral sessions (40). Parallel to the treatment of amphetamine psychosis, several drugs showed promise for the treatment of amphetamine dependence. Among them are methylphenidate, naltrexone, bupropion, sertraline, and mirtazapine, but the efficacy of these drugs is yet to be replicated (41).

### Objectives and Research Question

The treatment of amphetamine psychosis with antipsychotics underwent several investigations involving clinical trials, published case reports, letters to the editor, and brief reports. Clinical investigations suggested that antipsychotics could reduce or control the psychotic episode. The use of antipsychotics for the treatment of amphetamine psychosis is sparse due to complaints of disturbing side effects and adverse events. Some authorities disfavor the use antipsychotics, stating that the psychotic episode is self-limited. However, among individuals experiencing amphetamine psychosis, it may be difficult to rule out schizophrenia. These individuals may not fully recover from the psychosis and may develop full-blown psychosis, emotional, and cognitive disturbances. The psychotic episode presents an imminent risk for self-injury or injury to others. During the psychotic episode, amphetamine users may develop muscle breakdown, rhabdomyolysis, kidney injury, arrhythmia, strokes, and myocardial infarction. Antipsychotics seem to be necessary and clinically relevant. Focusing on controlled clinical trials, this review aims to investigate the clinical benefits and risks of antipsychotics for the treatment of amphetamine psychosis.

## Materials and Methods

### Study Design

A systematic review was conducted on antipsychotics studied for the treatment of amphetamine psychosis.

### Eligibility Criteria

**Figure 1:**
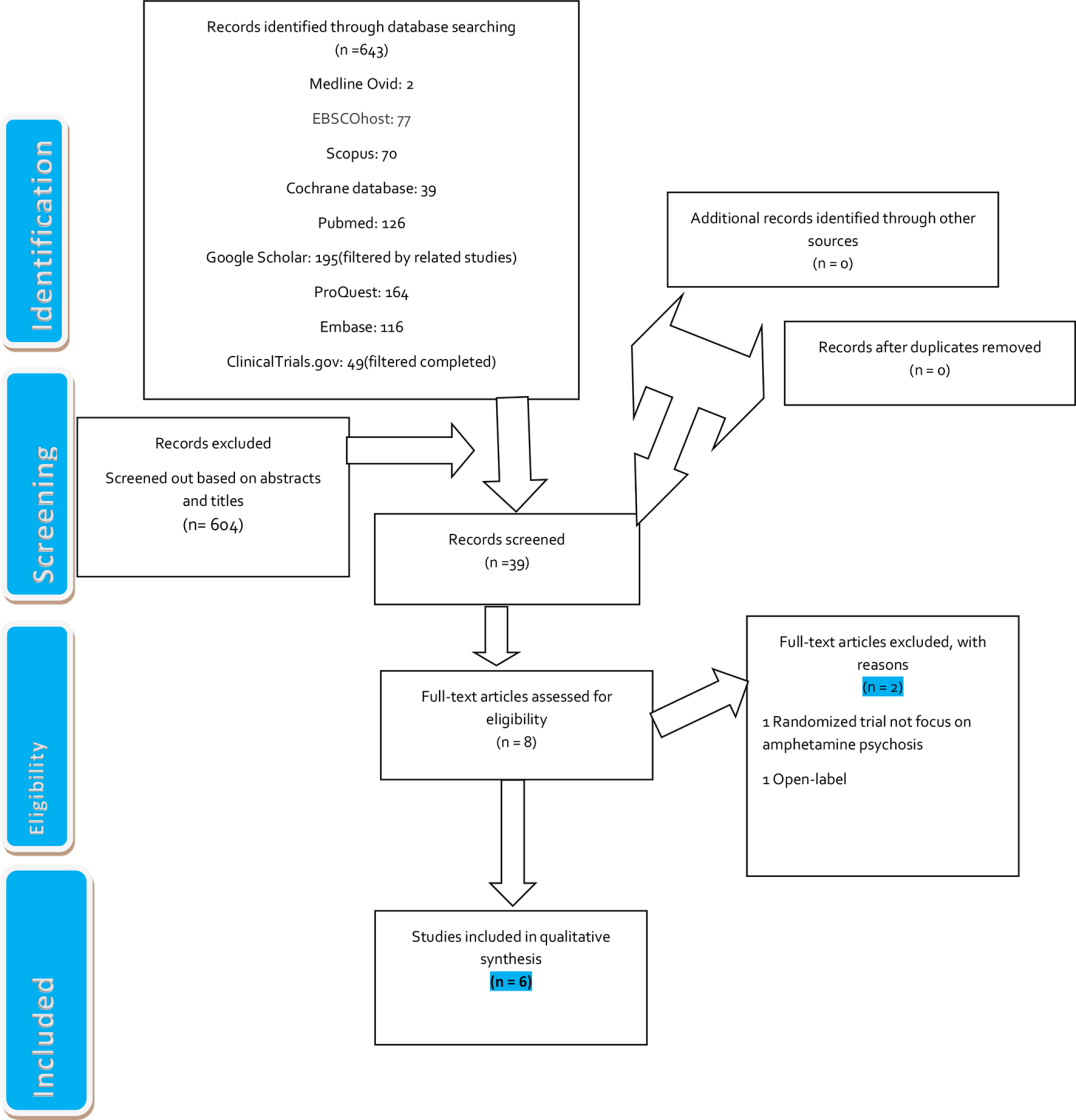
Flow diagram of the electronic search process. [Adapted from Moher D et al., (42).]

The search, screening, and selection process are in line with the Preferred Reporting Items for Systematic Reviews and Meta-Analyses (PRISMA) (**Figure 1**) (42). Ethical approval was not sought, because the review was a secondary analysis of anonymized data that were already published. Included studies were randomized controlled trials regardless of their language, year, or country of publication. Excluded studies were cross-sectional studies, longitudinal studies, uncontrolled trials, and non-randomized trials. The participants involved adolescent, middle-age, or older-age males or females. There were two settings: hospitals and outpatients.

### Search Strategy

Keywords “amphetamine,” “amphetamine psychosis,” “amphetamine-induced psychosis,” and “amphetamine psychosis-controlled trials” were free-texted into PubMed, Scopus, Google Scholar, EBSCOhost, ProQuest, Cochrane Review Database, Medline Ovid, and EMBASE (**Figure 1**). The same search technique was applied to ClinicalTrials.gov (U.S. National Library of Medicine) and the grey literature.

### Data Extraction, Quality Assessment, and Assessment of Risk of Bias in Included Studies

Two authors (DF and PM) separately searched for publications, screened abstracts, and retained potential articles for the review using the Abstraction Form (**Supplementary**). DF and PM collected data on study design, types of interventions, protocols, outcome measures, retention, dropouts, sample size, country of the study, setting, sex and age, funding disclosures, standardized scales, and data from tables. They separately scored the quality of the trials by using the Oxford Quality Scoring System (≤2: low range of quality score, ≥3: high range of quality score, and 5: highest score) (43) and granted a Grading of Recommendations, Assessment, Development, and Evaluations (GRADE) level of recommendation for each trial (44). The Cochrane risk-of-bias tool was used to assess selection, detection, attrition, and performance biases of individual trials (**Supplementary**) (45). A third author (KL) settled disagreements between the two authors.

### Types of Interventions

Experimental interventions involved interventions conducted in hospitals or outpatients involving an active drug for the treatment of amphetamine psychosis. Control interventions involved interventions conducted in hospitals or outpatients involving an active drug compared to another active drug or a placebo for the treatment of amphetamine psychosis.

### Types of Outcome MeasuresPrimary Outcomes

Efficacy: Clinical benefits defined by a reduction of psychotic symptoms measured by the Scale for the Assessment of Positive Symptoms (SAPS), the Scale for the Assessment of Negative Symptoms (SANS), the Positive and Negative Syndrome Scale (PANSS), and the Clinical Global Impression – Severity Scale (CGI-S). Risks: Side effects defined as an undesirable effect caused by the antipsychotics such as akathisia, agitation, hypotension, and sedation. Serious adverse events resulted (neuroleptic malignant syndrome) in death.

### Secondary Outcomes

Treatment retention, completion, and dropout.

### Measures of Treatment Effect

Data extracted included different types of raw data such as the mean score and standard deviation of outcome measures, the percentage of retention and dropout in a trial, and the average score of side effects or adverse events. For the effect size, when data provided the mean and standard deviation, we used the standardized mean difference between the two groups. An effect size of 0.2 was considered as having a small outcome, 0.5 was medium, and 0.8 was large. When data extracted could not be converted to the standardized mean difference, the effect size was reported in percentage.

### Assessment of Heterogeneity

The type of data collected was not combinable. We could only individually evaluate the studies included in the review; thus, we did not assess the data for heterogeneity.

### Data Synthesis and Analysis

Data collected on treatment benefits, side effects or adverse events, and retention to treatment were exported to an Excel spreadsheet in OneDrive and then converted to a format table. We created five subgroups of drugs studied for amphetamine-induced psychosis and synthesized the results in a qualitative format in the core manuscript, in addition to a quantitative format in two tables. The data we collected were not combinable. We were not able to perform a cumulative meta-analysis.

## Results

### Description of Studies

We identified 643 titles: PubMed, 126; Scopus, 70; Google Scholar (filtered by related studies), 195; ProQuest, 164; Cochrane Review Database, 39; Medline Ovid, 2; EMBASE, 116; EBSCOhost, 77; and ClinicalTrials.gov, 49 (filtered completed trials). We screened a total of 39 documents for the investigation after we removed 604 papers based on abstracts and titles. We selected eight full articles but excluded two other articles. [One randomized trial did not focus on amphetamine psychosis (46), and one trial was non-randomized (47) (**Figure 1**)].

### Risk of Bias in Included Studies

No trial was at high risk for selection bias. Two trials were at high risk of performance and detection bias due to insufficient or no description of blinding procedures. No trial was at risk for attrition or reporting bias. Overall, we judged that about 85% of the trials were at low risk of bias in general (**Supplementary**).

### Synthesis of Results

We reviewed six randomized controlled trials of participants (n = 314). Five trials compared an active drug to another active drug. Only one trial compared an active drug to a placebo. The age of the participants included was 18 and older, except for one trial in which participants were 15 and above (48). The mean average age of the participants was 32, with a standard deviation of 20.63. The number of participants per trials varied from setting to setting, with a mean average of 26.16 and a standard deviation of 7.61 (**Table 2**). Overall, there were more men than women. All the trials excluded participants with a history of an Axis I (schizophrenia, schizoaffective disorder) diagnosis based on the *Diagnostic and Statistical Manual of Mental Disorders* (*DSM*) (4th edition or 4th edition revised). One trial excluded participants with suicidal or homicidal ideation (49), and four trials excluded participants with intellectual disabilities (49–52). The trials also looked at follow-up data for a short period, and it is unknown whether the participants remained on antipsychotic medications for a long time or whether further episodes of psychosis developed. Most of the studies broke down participants’ characteristics by age, education, and marital status; however, this stratification did not appear to play a role in the outcome of this review. Around 67% of the trials received a Jadad score of 4, and none of the trials received a score of 2. (A trial that received a score of lesser than or equal to 2 is of low quality, and a score greater than or equal to 3 is of high quality.) For the six trials, we judged that further research is very likely to have a substantial effect on the confidence in the estimate of effect (benefit or risk) and is likely to change the estimate (**Table 2**).

**Table 2:** Provides a summary on demographic, setting, duration of a trial, study design, and standardized scales.

Study name	Participants	Setting/country	Age	Study design/duration	Jadad	GRADE	Drug doses (mg/d)	Standardized rating scales/amphetamine-induced psychosis criteria	Comments
Verachai et al. (51)	Quetiapine = 36	**Inpatient** hospital	**≥18**	**Randomized**	4	R+	Quetiapine: 100, 200, up to 300 mg/d	PANSS/	Double-blinded, methamphetamine-induced psychosis
	Haloperidol = 44	**Thailand**		4 weeks			Haloperidol: 2, 4, up to 6 mg/d	clinical interview and urine positive for methamphetamine	GRADE downgrades due to small sample size, no placebo controlled
Wang et al. (49)	Aripiprazole = 21	**Inpatient** hospital	18–60	**Randomized**	3	R+	Aripiprazole: 5–10 mg/d initially, 5–15 mg/d	PANSS/	Methamphetamine-induced psychosis
	Risperidone = 21	China		**25 days**			Risperidone: 2–4 mg/d initially, 4–6 mg/d	*DSM-IV* diagnosis criteria	GRADE downgrades due to small sample size, trial was not double-blinded
Farnia et al. (50)	Aripiprazole = 27	**Inpatient** hospital	18–60	**Randomized**	4	R+	Risperidone: 4 mg/d, bedtime	Assessment of negative symptoms (SANS) and	Double-blinded, amphetamine-induced psychosis
	Risperidone = 26	Iran		6 weeks			Aripiprazole:15mg/d, bedtime	assessment of positive symptoms (SAPS)/*DSM-IV* diagnosis criteria	GRADE downgrades due to small sample size, no placebo controlled
Sulaiman et al. (53)	Aripiprazole = 19	Medical center	18–60	**Randomized**	4	R+	Aripiprazole: 5–10 mg po daily	PANSS	Double-blinded and placebo-controlled, methamphetamine-associated psychosis
	Placebo = 18	**Malaysia**		8 weeks				*DSM-IV* diagnosis criteria	GRADE downgrades due to small sample size
Samiei et al. (52)	Haloperidol = 22	**Inpatient **hospital	35.3–	**Randomized**	3	R+	Haloperidol: 5 up to 20 mg/d	Scale of assessment of positive symptoms (SAPS)/	Not double-blinded, methamphetamine-associated psychosis
	Risperidone = 22	Iran	34.6	1 month			Risperidone: 2, 4, up to 8 mg/d	*DSM-IV-TR* diagnosis criteria	GRADE downgrades due to small sample size, no placebo controlled
Leelahanaj et al. (48)	Olanzapine = 29	**Outpatient**	**≥15**	**Randomized**	4	R+	Olanzapine: 5, 10, up to 20 mg/d	Brief Psychiatric Rating Scale	Double-blinded, amphetamine psychosis
	Haloperidol = 29	**Thailand**		4 weeks			Haloperidol: 5, 10, up to 20 mg/d	Clinical Global Impression Severity Scale/	GRADE downgrades due to small sample size, imprecision (broad 95% CI)
								*DSM-IV* diagnosis criteria	

Note that the table does not contain information on the results of individual trials (see **Table 3**). **Table 2** also provides a GRADE recommendation for individual study. Due to elevated heterogeneity, the GRADE was individualized per trial. The main reasons for a GRADE judgment of low were small sample size and limitations in study design.

The comments section provides the main reason for the GRADE recommendation.

Note that Jadad (Oxford Quality Scoring System) was calculated online with a new tool accessed at http://www.pmidcalc.org/?sid=8721797&newtest=Y.

GRADE, Grading of Recommendations, Assessment, Development, and Evaluations; METH, methamphetamine; DSM-IV, Diagnostic and Statistical Manual of Mental Disorders, 4th Edition; DSM-IV-TR, Diagnostic and Statistical Manual of Mental Disorders, 4th Edition—Text Revised; SANS, Scale for the Assessment of Negative Symptoms; SAPS, Scale for the Assessment of Positive Symptoms; R+, Further research is very likely to have an important impact on our confidence in the estimate of effect and is likely to change the estimate.

**Table 3:** Synthesis of the results in individual trial based on the three outcome measures.

Study name	Trial	Treatment response		Side effects, adverse events		Treatment retention, completion, dropout	
		Effect	95% CI				
Verachai et al. (51)	**Quetiapine** **Haloperidol**	SMD: 0.7602	0.2662–1.2542	**Hypotension** Quetiapine: 2.3%		**Dropout** Quetiapine: 5	
		Quetiapine reduced PANSS score					
		better than haloperidol		Haloperidol: 5.6%		Haloperidol: 7	
		SMD: 0.2914	0.1514–0.7341	***$*** **Sedation**		**Completers**	
		Cure rate: quetiapine was slightly		Quetiapine: 9.1%		Quetiapine: 31	
		superior to haloperidol		Haloperidol: 8.3%		Haloperidol: 37	
Wang et al. (49)	**Aripiprazole **	SMD: 0.6601	0.039–1.2812	**Agitation**		**Retention**	
	**Risperidone**	Days 1–7, aripiprazole reduced		Aripiprazole: 52.4%		Aripiprazole: 19.9 days	
		PANSS total score more than risperidone		Risperidone: 19.0%		Risperidone: 24 days	
		SDM: 0.0617	−0.5433–0.6667	**Anxiety**		**Discontinuation**	
		Days 10–16, risperidone had small superiority to aripiprazole in reducing PANSS total score		Aripiprazole: 57%		Aripiprazole: 33%	
		SDM: 0.6202	0.0009–1.2394	Risperidone: 29%		Risperidone: 0%	
		Days 1–7, aripiprazole reduced		**Akathisia**			
				Aripiprazole: 61.9%			
		CGI-S score more than risperidone		Risperidone: 28.60%			
		SDM: 0	−0.6049–0.6049	**Sialorrhea**			
		Days 10–16, no difference between both drugs in CGI-S score		Aripiprazole: 52%			
				Risperidone: 29%			
				**Dystonia**			
				Aripiprazole: 86%			
				Risperidone: 29%			
Farnia et al. (50)	**Aripiprazole ** **Risperidone**	SMD: −1	−1.6199–0.3801	No extrapyramidal symptoms No neuroleptic malignant syndrome		**Completion** Aripiprazole: 92%	
		Aripiprazole reduced SAPS score		No recorded sedation or akathisia		Risperidone: 88.6%	
		better than risperidone					
		SMD: −0.7746	−1.3806–0.1686				
		Risperidone reduced SANS score					
		better than aripiprazole					
Sulaiman et al. (53)	**Aripiprazole** **Placebo**	Psychotic symptoms decreased more among aripiprazole group more than placebo		**Akathisia** Aripiprazole: mean AIMS		**Dropout rate ** Aripiprazole: 83% after 27 days,	
				score: 26.6		82% after 58 days	
		#P < 0.05		Placebo: mean AIMS		Placebo: 66% after 27 days,	
				score: 5.6		44% after 58 days	
				**Agitation**			
				Aripiprazole: mean BARS			
				score:10.6			
				Placebo: mean BARS			
				score: 5.6			
				**Insomnia**			
				Aripiprazole: mean SAS			
				score: 10.6			
				Placebo: mean SAS			
				score: 11.1			
Samiei et al. (52)	**Haloperidol** **Risperidone**	SMD: 0.8895	0.0134–1.7655	No information or data reported		No information or data reported	
		Both drugs reduced methamphetamine psychosis					
		SMD: 0.2141	−0.3786–0.8067				
		Week 3: hallucination					
		Haloperidol had a small difference					
		over risperidone in reducing hallucination					
		Week 3: delusion					
		SMD: 0.5116	−0.0889–1.1122				
		Haloperidol had a moderate difference					
		over risperidone in reducing delusion					
		Week 1: bizarre behaviors					
		SMD: 0.6023	−0.0019–1.2065				
		Haloperidol had a moderate difference					
		over risperidone in reducing bizarre behaviors					
							
Leelahanaj et al. (48)	**Olanzapine** **Haloperidol**	SMD 0.912	0.3712–1.4528	**Somnolence**		**Completers**	
		End point score on Clinical Global Impression		Olanzapine: 15.4%		Olanzapine: 93.1%	
		Severity Scale and Brief Psychiatric Rating Scale in both groups		Haloperidol: 7.4%		Haloperidol: 65.5%	
				**Skin rash**			
		CGI score at end point		Olanzapine: 3.8%			
		Olanzapine > haloperidol (p = 0.37)		Haloperidol: none			
				**Headache**			
				Olanzapine: 7.7%			
				Haloperidol: none			

#P < 0.05 Based on data presented, SMD could not be calculated. The p-value is extracted from author’s text. $, based on the number of participants complaining of sedation. AIMS, Abnormal Involuntary Movement Scale; BARS, Brief Agitation Rating Scale; SAS, Simpson Angus Scale; CGI, Clinical Global Impression; SMD, Standardized Mean Difference.

### Clinical Benefits

#### Aripiprazole, Risperidone

Two trials studied aripiprazole and risperidone for amphetamine psychosis. One trial investigated risperidone dose of 4 mg daily at bedtime and aripiprazole 15 mg at bedtime for amphetamine-induced psychosis (50). Compared to baseline and at treatment completion, the paired-sample t-tests (p < 0.001) showed a reduction of psychotic symptoms in both the SAPS and the SANS. Investigation of the efficacy of the two drugs indicated that aripiprazole was statistically superior to risperidone at reducing poverty of speech, apathy, anhedonia, and inattentiveness. Risperidone was statistically superior to aripiprazole in reducing hallucination, delusion, bizarre behavior, and thought disorder. Overall, risperidone seemed to have better potentiality than aripiprazole in reducing positive symptoms of amphetamine-induced psychosis. Aripiprazole seemed to have better potentiality than risperidone in reducing negative symptoms. Another trial studied aripiprazole at a dose of 5–10 mg daily initially and 5–15 mg daily subsequently and risperidone at a dose of 2–4 mg daily initially and 4–6 mg daily subsequently for methamphetamine-induced psychosis (49). Analysis of data over 25 days indicated that both aripiprazole and risperidone produced a net reduction of psychotic symptoms on both the PANSS and the Clinical Global Impression Scale (CGI-S). Over time, results varied among the two groups. From days 1 to 7, aripiprazole showed superiority to risperidone in reducing the PANNS score; from days 10 to 16, risperidone showed a small superiority to aripiprazole (**Table 2**).

#### Aripiprazole, Placebo

One trial compared aripiprazole to placebo (53). Among subjects with methamphetamine dependence with psychosis, aripiprazole (5–10 mg) daily reduced the severity of psychotic symptoms on the PANSS scale (p < 0.005). Participants in the aripiprazole group showed a decrease in the CGI score. On the CGI-S, aripiprazole participants had an average score of 2, and for placebo, 2.1 (**Table 2**).

#### Haloperidol, Quetiapine

One double-blind, randomized controlled trial compared the efficacy of haloperidol to quetiapine for the treatment of methamphetamine-induced psychosis (51). Although the baseline score of psychotic symptoms in the haloperidol group was higher than that in the quetiapine group, at the study end point, and adjusted for demographic data, there was no significant difference between the antipsychotic effects of the two drugs measured by the mean total PANSS score. When considering the general estimating equations (GEEs), quetiapine appeared to reduce psychotic symptoms in a superior way to haloperidol (**Table 2**). The time to cure in both groups indicated a slight difference in favor of haloperidol.

#### Haloperidol, Risperidone

One trial compared haloperidol with risperidone for methamphetamine-associated psychosis (52). Over 4 weeks, in both haloperidol and risperidone groups, the SAPS was significantly reduced (p < 0.05). Several psychopathologic parameters (hallucination, delusion, and bizarre behaviors) were evaluated to establish the difference in the treatment efficacy of the two groups. From week 1 to week 3, haloperidol was superior to risperidone in controlling symptoms of hallucination, and for delusion and bizarre behaviors (**Table 2**).

#### Haloperidol, Olanzapine

Participants who exhibited symptoms consistent with amphetamine psychosis underwent a 4-week randomized double-blinded randomized trials treatment of olanzapine doses of 5, 10, and up to 20 mg daily and haloperidol doses of 5, 10, and up to 20 mg daily (48). In both groups, there was a significant improvement from baseline to end point measured by a reduction of the Clinical Global Impression Severity Scale and Brief Psychiatric Rating Scale scores (p < 0.001). At end point, there was a small difference in symptom reduction on the CGI-S in favor of olanzapine, but the result was not statistically significant (**Table 2**).

### Side Effects and Serious Adverse Events

None of the trials reported a life-threatening incident such as neuroleptic malignant syndrome.

#### Aripiprazole, Risperidone

In the trial of aripiprazole and risperidone for the treatment of methamphetamine-associated psychosis in Chinese participants, extrapyramidal symptom score based on the Simpson Angus Scale (SAS) and the Barnes Akathisia Rating Scale (BARS) increased in both groups, but there was no statistical difference between the two drugs. However, the incidence of agitation, anxiety, and akathisia was higher for aripiprazole comparing with risperidone (49). In a 6-week trial, Farnia et al. (50) reported that there was no complaint of extrapyramidal symptoms and no sedation or akathisia among participants allocated to aripiprazole or risperidone. No severe adverse event such as neuroleptic malignant syndrome occurred.

#### Aripiprazole, Placebo

The aripiprazole group developed more akathisia than placebo measured by the Abnormal Involuntary Movement Scale (AIMS) score and more agitation on the BARS score, but there was less complaint of insomnia in the aripiprazole arm than in the placebo arm (53).

#### Haloperidol, Quetiapine

There were no significant or life-threatening side effects reported. Data pointed out that haloperidol participants complained of less hypotension than quetiapine participants. Quetiapine participants complained of more sedation reported than haloperidol participants (51).

#### Haloperidol, Risperidone

In the trial of methamphetamine-associated psychosis and treatment with haloperidol and risperidone, haloperidol (5–20 mg/day) and risperidone (2–8 mg/day) doses were gradually titrated based on participants’ tolerance (52); there were no reported data on adverse events or side effects. It was unclear whether the gradual titration facilitated a better tolerance among the two groups.

#### Haloperidol, Olanzapine

Olanzapine participants complained of somnolence, headache, weight, and skin rash more than haloperidol participants. However, haloperidol participants developed extrapyramidal syndrome, hypertonia, dyskinesia, and hypersalivation more than olanzapine participants (48).

### Treatment Retention, Completion, and Dropout

#### Aripiprazole, Risperidone

Two trials compared aripiprazole with risperidone. One trial found that aripiprazole participants had a lower retention rate than risperidone participants (49), in contrast to another trial that favored aripiprazole over risperidone (50). The trial of aripiprazole versus placebo reported a higher dropout among aripiprazole participants on the 28th and 58th days (53).

#### Haloperidol, Quetiapine

Among participants completing the treatment, there was a slight difference in favor of quetiapine, with 86.11% completers and 84% for haloperidol (51). The most common reasons provided for dropout in both groups were: early discharge from the hospital, referral for medical treatment, elopement, and refusal to take medications.

#### Haloperidol, Risperidone

Samiei et al. (52) reported a randomized clinical trial that was designed and conducted in 2012. Participants seemed to tolerate both haloperidol and risperidone, but we could not find data on treatment retention.

#### Haloperidol, Olanzapine

From the beginning of the 4-week trial comparing olanzapine with risperidone for the treatment of amphetamine psychosis, more participants in the olanzapine group completed the treatment. At the beginning of the trial, two participants on haloperidol discontinued the treatment, and about one-third of the participants discontinued treatment because of extrapyramidal symptoms. A lack of efficacy of haloperidol or olanzapine was not among the reasons cited for treatment discontinuation (48).

## Discussion

### Summary of Main Findings

Aripiprazole, quetiapine, haloperidol, olanzapine, and risperidone appeared to effectively treat both positive and negative symptoms of amphetamine psychosis (amphetamine-induced psychosis or amphetamine-associated psychosis) with no significant adverse event. Among these drugs, there were different noticeable outcomes. Data of one trial showed that more aripiprazole participants remained longer in treatment than risperidone participants, although aripiprazole participants complained more of akathisia and agitation than risperidone participants. Moreover, aripiprazole was able to reduce amphetamine psychosis better than risperidone in two trials involving an active drug versus another active drug and in one trial that compared aripiprazole with placebo. Haloperidol was not superior to quetiapine in reducing symptoms of amphetamine-induced psychosis. Quetiapine caused more hypotension but less sedation than haloperidol. Haloperidol seemed to be superior to risperidone in managing amphetamine psychosis. There was no significant difference between haloperidol and olanzapine. Both groups showed significant improvement of psychotic symptoms from baseline to end point. Overall, aripiprazole, haloperidol, quetiapine, olanzapine, and risperidone are effective and safe for the treatment of both positive and negative symptoms of amphetamine psychosis. This study agrees with a 2008 review in which both olanzapine and haloperidol were suggested to be efficacious in resolving amphetamine-induced psychotic symptoms (54); however, the two drugs were different in terms of their side-effect profile.

#### Psycho-Pharmacological Characteristics of Aripiprazole, Quetiapine, Haloperidol, Olanzapine, and Risperidone and Their Possible Mechanisms on Positive and Negative Psychotic Symptoms in Amphetamine Users

The neurobiological mechanism of action of antipsychotics on positive and negative symptoms in amphetamine users experiencing psychotic symptoms is yet to be elucidated. One of the challenges of antipsychotics is that beyond dopamine (DA) or serotonin, antipsychotics can also modulate multiple other neuroreceptors to produce different clinical results. The mechanism of action of aripiprazole is associated with the drug partial dopaminergic agonist’s activity (postsynaptic dopamine2 receptors and presynaptic autoreceptors). Aripiprazole also displays partial agonism at serotonin1A receptors and antagonism at serotonin2A receptors. Definitive advantages associated with DA partial agonism have yet to be determined (55). Aripiprazole occupies approximately 95% of dopamine D2 receptors in the striatum (56, 57). Aripiprazole’s ability to block the acute stimulatory effects of amphetamine and its possible positive effect on the decreased number of dopamine D2 receptors during drug dependence (58) may explain its favorable benefit at reducing negative psychotic symptoms. Risperidone is a monoaminergic antagonist with high affinity for the serotonin (5HT2), DA (D2), a1 and a2 adrenergic, and histaminergic (H1) receptors. In a study that examined the effects of risperidone and clozapine on amphetamine-induced striatal DA release in patients experiencing psychosis, amphetamine-induced striatal 11C-raclopride binding changes were not affected by treatment with clozapine or risperidone. The authors recommended further studies to examine the effects of risperidone and clozapine as well as other antipsychotics on the central DA functions, serotonergic, cholinergic, and glutamatergic, and even on DA receptor subtype in clinical populations (59). Via the caudate nucleus, haloperidol inhibits the ascending reticular system, competitively blocks the DA receptor (postsynaptic) in the mesolimbic system, and increases the turnover of brain DA. Haloperidol also blocks the adrenergic receptor and inhibits NMDA responses (60–62). Xue et al. (63) found that haloperidol was ideal in improving psychotic symptoms but did not significantly improve anxiety and depression. One can extrapolate that haloperidol may be more efficacious in managing positive psychotic symptoms, but the mechanism underlying this benefit needs further study. Quetiapine has affinity for the serotonin 5HT1A and 5HT2 receptors as well as dopamine D1 and D2 receptors. The drug also has a high affinity at the histamine H1 receptors (64). Quetiapine attenuated the dl-amphetamine–induced hyperthermia and the anxiety-like behavioral changes in rats. He et al. (65) suggested that quetiapine could normalize the induced anxiety-like behavioral by dl-amphetamine *via* either DA or serotonin. Quetiapine’s efficacy was comparable with haloperidol’s efficacy on the PANSS score in the trials reviewed. The benefit of antipsychotics for amphetamine psychosis (negative or positive symptoms) may be attributed to other receptors beyond the DA receptors. Olanzapine binds with high serotonin (5HT2A/2C, 5HT6), DA (D1–4), histamine (H1), and adrenergic (a1) receptors, where it acts as an antagonist. It also binds with serotonin (5HT3) and muscarinic M1–5 receptors. A study suggested that olanzapine could have a neuroprotective benefit against methamphetamine-induced cell deaths. Pretreatment with olanzapine reduced methamphetamine-induced mortality and hyperthermia in rats and counteracted the decrease of TH and B-cell lymphoma 2 (Bcl-2) immunostaining in the caudate–putamen. Olanzapine may protect rats against the dopaminergic terminal damage in the caudate–putamen (66). The benefit of olanzapine for amphetamine psychosis may rely on the drug’s potentiality to decrease cell death.

Finding the principal receptor target of antipsychotics is challenging in schizophrenia. Finding the main receptor target seems to even be more challenging in amphetamine psychosis.

#### Balancing Risk and Efficacy of Atypical/Typical Antipsychotics

Although the five drugs reviewed seem to be effective in managing amphetamine psychosis, balancing their risks and benefits must be individualized. Only haloperidol is a first-generation “typical” antipsychotic. The metabolic side-effect profile of the second-generation “atypical” antipsychotics such as weight gain, hyperlipidemia, diabetes mellitus, and diabetic ketoacidosis may limit their use in some patients. Besides, electrolyte and electrocardiogram (EKG) abnormalities, as well as cardiovascular adverse events, may require a solid baseline and periodic medical risk assessment (57). Amphetamine users are vulnerable to dehydration, acute kidney injury, and arrhythmia. Fornaro et al. (67) suggested that the ideal “antipsychotic agent” (in bipolar disorder, for example) may not only be efficacious based on the acuity of the clinical presentations but also be efficacious in the prevention of the psychotic episode throughout the illness. The trial with the most prolonged duration lasted 8 weeks (53); none of the trials followed up the participants over time. Balancing a long-term risk–benefit of the atypical in the trials reviewed can only be inferred from studies in schizophrenia. The ability of the atypical in preventing further psychotic episodes in users of amphetamine needs research. Haloperidol carries an elevated risk of extrapyramidal syndrome. However, a drug like risperidone also carries the risk of extrapyramidal syndrome at a high dose. A common single nucleotide polymorphisms (SNP) (rs167771) in the DRD*3* gene was suggested to be a candidate gene for risperidone-induced extrapyramidal syndrome (68). Clinton et al. (69) found that haloperidol was safe and efficacious in the management of agitation in the emergency setting. There are other “typical” antipsychotics besides haloperidol that may also be efficacious in managing agitation in the emergency setting. Inhaled loxapine has a relatively rapid onset of action, and it was shown to be efficient and well tolerated for agitated patients with schizophrenia and bipolar disorder (70). The risk of bronchospasm and the method of administration of loxapine (full patient collaboration) may hamper the use of loxapine in the emergency setting. In a 12-week randomized clinical trial, risperidone, haloperidol, and olanzapine showed a worsening lipid profile (total cholesterol and low-density lipoprotein cholesterol levels). Olanzapine group showed a significant increase in triglyceride levels (71). Aripiprazole may cause less or no metabolic syndrome (obesity, hypertension, or dyslipidemia). However, such advantage varies from individual to individual depending on premorbid medical risks and genetic predisposition. Analysis of data from the 4th Korean National Health and Nutrition Examination Survey found that male Koreans with schizophrenia who used aripiprazole, olanzapine, or risperidone for more than 3 months were more likely to develop metabolic syndrome than the general population (72). Clinicians should balance the benefits and risks of antipsychotics for individuals who experience amphetamine psychosis. These individuals are maybe more vulnerable to life-threatening cardiovascular events, neurocognitive deficits, and severe kidney injury.

#### Consideration of the Use of Long-Acting Antipsychotics

Should we consider the use of a long-acting antipsychotics for amphetamine psychosis? The trials reviewed did not follow-up the participants over a long time period. Compliance with treatment over a long period will most likely be a significant challenge. Another factor is the vulnerability of individuals with drug addiction and dependence on life-threatening medical complications. A long-acting antipsychotic may help control the psychotic symptoms and may prevent further damage to the brain, but such recommendation must be taken with caution because there is much that needs to be known about the psychopathology of amphetamine psychosis. One again, balancing the risks and benefits of antipsychotics for amphetamine psychosis is an essential step to consider before one can decide to treat amphetamine psychosis with an antipsychotic.

### Limitations

The trials included in this review provided information on treatment benefit, safety risk, completers, dropouts, and retention in treatment. The sample size was small, and the number of trials was also small. We could only find one trial that compared an active drug with a placebo. The other trials compared a known active agent that could also be used for the treatment of amphetamine psychosis. There was limited evidence on how an active agent versus another agent could individually treat amphetamine psychosis, knowing that these agents can treat psychosis of different etiologies other than “substance-induced psychosis.” Aripiprazole, quetiapine, haloperidol, olanzapine, or risperidone could treat the psychotic episode whether amphetamine was the culprit or not. A plausible explanation is that aripiprazole, for example, as a partial agonist of DA (D2), could have probably resulted in better control of amphetamine psychosis based on studies suggesting that amphetamine exerts its rewarding and reinforcing effects by elevating extracellular DA (73). This analysis contradicts the finding that haloperidol, which belongs to the DA class, a D2 receptor antagonist, was found to be efficacious for the treatment of amphetamine psychosis. Besides, aripiprazole is not strictly a DA agonist but also a 5HT1A agonist and a 5HT2A antagonist. There are several other neurobiological explanations inherent to antipsychotics that have more affinity for the DA receptor than the serotonin receptor or vice versa; their interplay and difference are still debatable. Such limitations are barriers to being able to predict treatment benefit or side effects accurately. The specificity of the ability of certain antipsychotics to treat amphetamine psychosis is uncertain. Other limitations are that some side effects reported of the five drugs investigated in this review are common side effects observed in the treatment of primary psychotic illnesses. Thus, the side effects presented here may not be specific to amphetamine psychosis. 

## Conclusions

The delineation of neurophysiological mechanisms that underlie different psychotic disorders (schizophrenia, schizoaffective disorder, or substance-induced psychotic disorder) is still ongoing. Besides probing genetics, neurotransmitters, and brain imaging as biomarkers, electroencephalography (EEG) has been proposed as one of the methods to portray a psychotic disorder. Howells et al. (74), in a case-controlled study of participants in outpatients from the Western Cape Province, South Africa, found that delta/alpha frequency activity during resting with eyes closed was lower in the control group compared with participants diagnosed with schizophrenia and methamphetamine-induced psychotic disorder, and lower for bipolar disorder than methamphetamine-induced psychotic disorder. Similar results were reported during the resting with eyes open and continuous performance task (74). Sato (75) suggested that a lasting change at the nerve terminal membranes’ transporters in the striatum and nucleus accumbens may provoke the induction and expression of stimulant-induced sensitization, which may engender vulnerability to schizophrenia-like psychotic episodes in methamphetamine-induced psychosis. Single-photon emission computed tomography showed a reduction in DA transporter density in the nucleus accumbens and caudate/putamen associated with the duration of methamphetamine use. Magnetic resonance spectroscopy showed a reduced ratio of creatine plus phosphocreatine (Cr + PCr)/choline-containing compounds (76). These findings support that amphetamine psychosis is a different clinical entity. Pharmacological treatment for amphetamine psychosis may need to be specific to the disease itself.

This review suggests that antipsychotics are efficacious and clinically relevant for the treatment of amphetamine psychosis. However, amphetamine psychosis may respond to any of the arsenal of antipsychotics for the treatment of primary psychotic illnesses (psychosis not induced by a drug), and the psychosis induced by amphetamine may be self-limited. The cause of the disease is amphetamine, but the effect is difficult to delineate. A more targeted treatment will perhaps be found when neurophysiological findings can explain the clinical manifestation of the illness.

Individuals with amphetamine psychosis are both medically and psychiatrically unstable. The possibility exists that the psychosis may become chronic and trigger the development of a full-blown primary psychosis in patients who are genetically predisposed to a primary psychotic illness. Thus, the choice of treating amphetamine psychosis with antipsychotics seems to be clinically and ethically appropriate. Practitioners need to tailor their use based on individuals’ risks for side effects.

The trials investigated lacked placebo-controlled arms. The feasibility of a placebo-controlled trial in mentally unstable patients is challenging for researchers due to ethical and legal problems that may surface. It is not clear, considering these ethical limitations, whether more placebo-controlled trials are needed for the treatment of amphetamine psychosis.

## Author Contributions

DF and PM separately conducted the literature search, extracted, and analyzed the data. DF and PM separately scored the quality of the trials included in the review. DF developed the tables and figures. DF and PM summarized the results. KL played the role of moderator, proofread the manuscript, proposed word choice, and grammatically corrected sentences. KL also contributed to gathering and formatting the list of references. DF and PM wrote the manuscript.

## Conflict of Interest

The authors declare that the research was conducted in the absence of any commercial or financial relationships that could be construed as a potential conflict of interest.
