# Supplementary material for: Antipsychotics for Amphetamine Psychosis. A Systematic Review
Source: Front Psychiatry. 2019 Oct 15;10:740. doi: 10.3389/fpsyt.2019.00740 (PMC6804571; doi:10.3389/fpsyt.2019.00740)

Data Abstraction Form

Introduction

This data abstraction form is a standard instrument used to systematically collect data from scientific reports in development of the Guide to Community Preventive Services (the Guide). You and one other abstractor will review each paper by using this form. After members of the chapter development team reconcile any differing responses, the data from these forms will be used in evidence databases and tables. Recommendations in the Guide will be based on this evidence. The data required to fill in the form will provide information on the intervention under study, evaluation setting and study population, outcomes, results, and study quality.

The three sections of the form consist of **Part I. Classification Information**, which is filled out by the chapter development team and reviewed and edited by the abstractors; **Part II. Descriptive Information** about the intervention, evaluation study characteristics, measurement of outcomes, and results; and **Part III. Study Quality** about the execution of the study. On average, it should take you 2 to 3 hours to read a paper and fill out the form. A note on formatting: Some of the questions are included in tables or text boxes. These boxes are included to ease readability of the form, NOT to limit the amount of information you can provide. If you need additional space, feel free to use the margins, other available space, or additional pages to write your answers. Also note that only the data sheets are to be completed; the instruction pages provide details and examples to help you respond to questions on the facing data sheet pages.

We have provided examples and commentary in italics throughout the instructions to help you do consistent reviews. However, if any questions arise during your reviews, feel free to contact the chapter development team to clarify any issues that are confusing.

To return the form, fax **ONLY this cover sheet and the pages labeled "Data Sheet" in the upper right hand corner (i.e., the right-hand pages)**. Note: If you elect to mail rather than FAX your forms, copy the cover page and the data sheets and mail those to us; retain the original form in your files until the chapter development team has contacted you to review the evidence tables and reconcile any differences from those of the second abstractor.

Notes:
1. For all multiple-choice questions, checking more than one response is acceptable and appropriate.
2. Indicate page or table numbers where data are located in the paper to aid checking the information.

Tracking Information:

Topic:

Subtopic:

Intervention title:

Reviewer Name:

Tracking Number:

Citation:

Study type:

☐ Published article	☐ Abstract/presentation
☐ Technical report	☐ Book/book chapter
☐ Unpublished dissertation/thesis	☐ Other *Specify:*

INSTRUCTIONS

Part I. Classification Information

For questions 1-3, review carefully the information provided and check the appropriate box in the gray shaded area to indicate if the chapter development team's assessment is correct or incorrect. If the assessment is incorrect, provide the correct information.

1. Study Design: See figure on back page: "Study Design Algorithm"
2. Intervention Components: Many interventions have more than one component. Check all that apply.

<u>Provision of information only</u>: These interventions try to change knowledge, attitudes or norms. Intervention methods might involve instruction (e.g., classes, assemblies), small media (e.g., brochures, leaflets, posters, letters, newsletters) or large media (e.g., television, radio, newspapers, billboards). For these interventions, also note the target population.

<u>Behavioral interventions</u>: These interventions try to change behaviors by providing necessary skills or materials. Intervention methods might involve modeling or demonstration, role playing, participatory skill development, individual benchmarking (i.e., goal-setting and achievement), providing feedback, providing incentives or penalties, or providing materials necessary to perform the desired behavior (e.g., condoms, car seats). For these interventions, also note the target population.

<u>Environmental interventions</u>: These interventions try to change the physical and or social environment to promote health or prevent disease. Interventions in the physical environment might involve adding to (e.g., fluoride in water systems), changing (e.g., resilient playground surfaces), or subtracting from (e.g., lead in gasoline and paint) the environment. Interventions in the social environment might include increasing employment opportunities (e.g, welfare-to-work programs) or development of community coalitions to change social systems (e.g., Detroit's "Angel's Night" anti-arson program).

<u>Legislation/Regulation/Enforcement</u>: These interventions try to change behaviors or alter disease risk factors by legislating particular behaviors, regulating risk factors, and enforcing those laws and regulations.
Examples:
- *Mandatory seat belt use laws*
- *School vaccination laws*
- *Increasing tobacco taxes*

Clinical: These interventions aim to increase access to and assurance of clinical care (patient-focused).

Public health or medical care system interventions: These interventions aim to change the public health or clinical care systems to increase or improve delivery of services (system-focused).
Examples:
- *Development of registries and surveillance systems*
- *Incentives to develop hospital policies for standing orders for vaccine administration*

2b. Was the intervention part of a larger intervention effort?
Example: a school based anti-drug educational program was implemented as a segment of a multi-state comprehensive health risk behavior modification program and is evaluated in this study.

3. Primary Outcome Measure(s): How was (were) the outcome measure(s) defined? Check all that apply and provide the definition used by the authors.
- Behavior (e.g., *observed correct use of child-restraint devices by children aged.≤ 5 years)*
- Other intermediate or mediating outcome: an outcome that precedes or is correlated with one or more health outcomes and stems from exposure to a determinant (e.g., *possession of child-restraint devices)*
- Non-fatal health outcome (e.g., *non-fatal motor vehicle occupant injury rates among children aged. ≤ 5 years).*
- Severity of illness/injury *(e.g., injury severity scores among children ≤ 5 years injured in motor vehicle crashes).*
- Death *(e.g., fatal motor vehicle occupant injury rates among children. ≤ 5 years).*
- Surrogate outcome: an outcome that is considered to be a proxy for health or other outcomes of interest (e.g., *number of citations issued for non-use of child-restraint devices when required by law).*

Part I. Classification Information

DATA SHEET

1. Study Design:

☐ Randomized trial (experiment)
  ☐ Individual ☐ Group
☐ Non-randomized "trial" (with ≥1 comparison group)
  ☐ Individual ☐ Group
☐ Prospective cohort study
☐ Other designs with concurrent comparison groups
☐ Retrospective cohort study
☐ Case-control study
☐ Time series study
☐ Before-after study
☐ Cross-sectional study
☐ Non-comparative study
☐ Other *Specify:*
☐ The study design indicated by the chapter development team is correct.
☐ The study design indicated by the chapter development team is incorrect or insufficient. I have added to or corrected the above information.

2. Intervention Components: *(Check all that apply)*

☐ Provision of information only ☐ General ☐ High-risk group ☐ Professional group
☐ Behavioral intervention ☐ General ☐ High-risk group ☐ Professional group
☐ Environmental intervention ☐ Physical environment ☐ Social environment
☐ Legislation/Regulation/Enforcement
☐ Clinical
☐ Public health or medical care system intervention
☐ Other *Specify:*
☐ This paper does not evaluate an intervention.
☐ The intervention components indicated by the chapter development team is correct.
☐ The intervention components indicated by the chapter development team is incorrect or insufficient. I have added to or corrected the above information.

2b. Was the intervention part of a larger intervention effort?

☐ Yes *(describe in Part II, question 1)*
☐ No

3. Primary Outcome Measure(s)

☐ Behavior *Describe:*
☐ Other intermediate or mediating outcome *Describe:*
☐ Non-fatal health effect *Describe:*
☐ Severity of illness/injury *Describe:*
☐ Death *Describe:*
☐ Surrogate Outcome *Describe:*
☐ The outcome measure(s) indicated by the chapter development team is (are) correct.
☐ The outcome measure(s) indicated by the chapter development team is (are) incorrect or insufficient. I have added to or corrected the above information.

Part II. Descriptive Information

A. Description of the Intervention

1. Use the following parameters to describe the intervention. The requested information might not be reported in the paper; if so indicate whether it is "Not available" or "Not applicable." Provide as much of this information as possible, and **include other relevant aspects of the intervention as necessary**.

- ***What is the proposed intervention?*** Describe the level or scale of focus (i.e., individual, family, group, community, or general public). Describe the services, materials, and other information that were delivered, or the policy or law that was enacted (include information about enactment, implementation, and enforcement).
- ***How is the intervention being delivered?*** Describe who delivered the intervention (e.g., health professional, volunteer, peer), how they were trained, and how they were assigned. Describe how the target population learned about the intervention. Describe the time period, frequency, and duration of the intervention. Describe the scope of the intervention (i.e., how many members of the target group(s) were reached by the intervention). Describe the extent of coordination with other agencies/organizations and the target community.
- ***Who is being targeted?*** Again, this might be broader than the population that was studied in the evaluation; briefly describe the characteristics of the target population.
- ***Where is the intervention being delivered?*** The intervention might be delivered in a particular type of setting or community-wide. This parameter should be described for the intervention as it was implemented, which might be in a setting broader than that which was studied in the evaluation.

Examples:

- ***What:*** *Individual parents received 1 or 2 personalized postcard reminders signed by their pediatrician to remind them their children were due for MMR vaccination and to present the adverse health consequences of being unvaccinated. If no response was made to postcard reminders, a public health nurse made up to 3 attempts to visit the family at their home to provide vaccination;* ***How:*** *pediatricians and other office staff, monthly from 1986-88, all patients in 4 practices;* ***Who:*** *parents of children aged 0-2 years;* ***Where:*** *4 pediatricians' offices in southern California and patient homes.*
- ***What:*** *Community-level (county) policy for surfaces of intervention playgrounds to be covered with pine straw to a depth of 6" in a radius of 9'from every piece of climbing equipment;* ***How****: county school board policy instituted on 7/1/83;* ***Who:*** *children aged 3-9 years;* ***Where:*** *all pre-K through elementary schools in 1 county.*
- ***What:*** *Community-level (state) requirement that families provide documentation signed by physician that children received at least 5 doses of DTP vaccine, 4 doses of OPV vaccine, and 1 dose of MMR vaccine prior to kindergarten entry. Children without such documentation were prohibited from attending school;* ***How:*** *state law enacted 4/1/82; enforcement began 8/1/82;* ***Who:*** *parents of children entering kindergarten;* ***Where:*** *Ohio.*
- ***What:*** *Community-level (school) 10-hour education program for resistance to drug use. Curriculum included information about consequences of drug use, correction of beliefs about the prevalence of drug use, counteraction of community norms promoting drug use, practice of pressure resistance, and a public commitment to avoid drug use. Teaching methods included peer teaching, modeling and role playing, and feedback and peer involvement. Course content approved by local PTA and police;* ***How:*** *peers (12th grade students with 4 hours of training) and professional health teachers, required class offered each of 3 terms, 1994-95 school year;* ***Who:*** *12th grade students;* ***Where:*** *John Doe High School, Peoria, Illinois.* ***Other:*** *program implemented as part of a multi-state, multi-component health risk behavior modification program.*

2. Did the authors describe the formative research, theoretical basis(es), or construct(s) upon which the intervention was developed? If so, provide as much information as necessary to identify the relevant theory.

3. What type of organization implemented the intervention (i.e., directly interacted with the population under study, not organizations that might have provided scientific or financial support)? Check all that apply.

4. Describe any intervention(s) deliberately or inadvertently applied to the comparison or control group(s). **Indicate the page where this information is found.** If the study did not have a comparison or control group, or had a comparison group to which no intervention was applied, skip to question 5.

Examples:

- *Families in the comparison group received usual care, which involved a 1-page handout written in English or Spanish describing the potential hazards of deteriorating lead-based paint.*
- *Families attending the comparison clinic did not receive an intervention as part of the study; however, in Minneapolis and unrelated to the study, during the study period weekly 30-second televised public service announcements were aired encouraging women aged 40 years and over to have annual mammography.*

Part II. Descriptive Information

DATA SHEET

A. Description of the Intervention

1. *What:*

How:

Who:

Where:

Other:

2. Theory described?
☐ Yes *Describe:*
☐ No

3. Type of organization *(Check all that apply)*
☐ Managed care organization
☐ Other clinical organization
☐ Academic organization
☐ Community-based organization
☐ Other *Specify:*
☐ Unknown
☐ Public health agency: ☐ Federal ☐ State ☐ Local
Specify:
☐Other governmental agency: ☐ Federal ☐ State ☐ Local
Specify:
☐ Does not apply

4. Interventions for a comparison or control group(s):
☐ No comparison group
☐ No intervention for comparison group (purposefully or inadvertently)
☐ Intervention applied to comparison group *Describe:*

B. Evaluation Study Characteristics
(These questions refer specifically to the setting and population that were studied in the evaluation of the intervention.)

Place and Time
5. Location: Where was the **study** done? Specify the city, state, region, etc.

6. Population density: Was the **study** done in an urban, suburban, or rural setting?

- Check the appropriate box AS DESIGNATED BY THE AUTHORS.
- Check **"Mixed"** ONLY if the intervention was applied to the entire population of a large geographic area that likely covers urban, suburban, and rural settings.
- If the authors do not state the population density but do provide ancillary information that allows you to make that determination (e.g., population size, description of the setting, and other community characteristics), use your best judgment to check one of the boxes.
- If you are unsure about the population density, but the authors report the population size or other information, include that information in the margin without checking one of the boxes.
- Check **"Not reported"** if the authors do not provide sufficient information about the community to determine the population density.

7. What was the setting in which the intervention was implemented for the purposes of conducting the study? Check all that apply. This might be the same as or a subset of the settings in which the intervention was implemented as described in Part II/Question 1.
Examples:

- *Legislation was implemented state-wide. Check "community-wide" and write in "state."*
- *An intervention was implemented in schools, shopping malls, and worksites throughout a county. The evaluation of its effectiveness, however, was limited to the schools. Check "schools."*

8. How were outcome and other independent (or predictor) variables measured? Check all that apply. See Part I/Question 3 for relevant outcome measures. **Provide information on observer or interviewer training and blinding, as well as inter-observer agreement as appropriate.**

Response Option	***Examples*** **or Definitions**
**Resource utilization**	*Hours of media exposure or number of reminders distributed*
**Observation**	Self-explanatory
**Interview**	Telephone or in-person interview
**Self-administered questionnaire**	Any written questionnaire that is completed by study participants
**Laboratory test**	*Serum or urine drug levels to assess compliance with drug therapy*
**Record review**	Self-explanatory
**Other**	
**Not reported/Did not assess**	Self-explanatory

Example: *The study reported observed correct use of child-restraint devices, using trained but unblinded observers. Inter-observer agreement was performed. Check the response option "Observation" and provide the following: "Correct use defined as child-restraint device tethered to automobile seat with child appropriately harnessed. Observers trained, not blinded. Inter-observer agreement for use= 93% and for estimated age of the child= 83%, k= .76 and .64 respectively."*

9. Where were outcomes and other variables assessed? If this was the same as the intervention setting, check "same." If different, describe using the same categories as in Part II/Question 7.
Example: *The intervention was implemented in clinics, but measured at observation sites throughout the community. Check "Different from the intervention setting" and write in "community-wide."*

10. Over what time period (include dates) and at what intervals were outcomes and other variables measured?
Example: *The study measured self-reported smoking behavior at 3-month intervals for 2 years after the intervention from January 1986 to December 1987.*

Part II. Descriptive Information

B. Evaluation Study Characteristics
Place/Time

5. Location:
☐USA
☐ Other industrialized country
☐ Developing country

6. Population density *(Check all that apply)*
☐ Urban ☐ Suburban ☐ Rural ☐ Mixed ☐ Not Reported

7. Setting *(Check all that apply)*
☐ Hospital
☐ Clinic or health-care provider office
☐Nursing home
☐ Child day care center
☐ Drug treatment facility
☐Mental health setting
☐Community-based organization
☐School
☐Workplace
☐Religious institution
☐ Home
☐ Prison
☐ Street
☐ Shelter
☐ Community-wide
Describe:

☐ Other setting *Specify:*
☐ Does not apply

8. How were outcomes and other independent (or predictor) variables measured?
☐ Resource utilization
Describe:

☐ Observation
Describe:

☐ Interview
Describe:

☐ Self-administered questionnaire
Describe:

☐ Laboratory test
Describe:

☐ Record review
Describe:

☐ Other
Describe:

☐ Not reported/Did not assess

9. Where were outcomes measured?
[ ] Same as intervention setting
[ ] Different from intervention setting *Describe:*

10. Time period and intervals outcome(s) measured

INSTRUCTIONS

Part II. Descriptive Information

Person (Study Population) (i.e., intervention and comparison populations)
See instructions for question 14 to differentiate the study population from other groups for whom demographic information or results might be reported in the paper.

11 a. Describe the eligibility criteria required to enter into the study population.

11 b. **For studies in which the investigator allocated** subjects to intervention/comparison groups, describe the groups or individuals who were allocated and the total number eligible for inclusion in the study (N =sampling frame). *Of those eligible*, provide the numbers of groups/or individuals who were allocated. Also provide descriptions of the groups or individuals who were observed and included in analyses and provide the numbers of groups or individuals who were observed and included in analyses. For observational studies in which the investigators did not allocate intervention and control conditions, describe the groups or individuals who were observed and included in the analysis; enter NA in the allocation columns for these studies. Many study designs have samples selected or make measurements at multiple points in time; include this information if it is provided. (See first example, below.)

Use the following sampling codes in the columns headed "Samp." under Allocation and Observation:

E = Entire eligible population — **NR =Not reported**
P =Probability sample — **NA =Not applicable**
C = Convenience/self-selected sample

Example: *One community received a child-restraint-device distribution program through the community clinic. Neighboring community: no intervention. Mothers of all eligible children in each community interviewed regarding child-restraint-device use when children 3 and 13 months old· all individuals with complete data were included in analysis of the 2 groups.*

	Description of groups or individuals N = sampling frame	Allocation				Observation				Number Analyzed
		Intervention		Comparison		Intervention		Comparison		
		n	Samp	n	Samp	n	Samp	n	Samp	
**Groups 1**	Communities N = undefined	1	C	1	C	1	E	1	E	2
**2**										
**3**										
**Individuals**	Child MV occupants<br>3 mo. N = 635<br>13 mo. N=510					336<br>276	E<br>E	214<br>182	E<br>E	

Example: *Investigators conducted a time series analysis on all reports of child motor vehicle crash injuries from a state-wide accident reporting system from 1979 through 1986; a mandatory child-restraint-use law was enacted in 1983. Data were analyzed for all children identified in the database with complete information about injuries and restraint use.*

	Description of groups or individuals N = sampling frame	Allocation				Observation				Number Analyzed
		Intervention		Comparison		Intervention		Comparison		
		n	Samp	n	Samp	n	Samp	n	Samp	
**Groups 1**		NA		NA						
**2**										
**3**										
**Individuals**	Injured children N = 10,132					5,021	E	5,111	E	10,132

Example: *Investigators randomly allocated all 50 clinics serving high-risk populations in a community (of 150 total clinics serving all populations) to either intervention or comparison groups. Intervention clinic physicians were provided with an educational intervention designed to improve vaccination rates. Because of resource constraints, 5 randomly selected clinics in each group were observed for results and analysis. Vaccine coverage was collected from individual patient charts; coverage rates were calculated for the two groups overall (intervention vs. comparison) and grouped by clinic and attending physician.*

	Description of groups or individuals N = sampling frame	Allocation				Observation				Number Analyzed
		Intervention		Comparison		Intervention		Comparison		
		n	Samp	n	Samp	n	Samp	n	Samp	
**Groups 1**	Clinics, N=50	25	E	25	E	5	P	5	P	2 Int v Com 10 clinics
**2**	Physicians, N=22					10	E	12	E	22
**3**										
**Individuals**										

Part II. Descriptive Information

DATA SHEET

Person (Study Population)
11 a. Eligibility criteria: *Describe:*

11 b. Levels of allocation, observation, and analysis: description and numbers of groups and individuals and methods of sampling. (See instructions for sampling codes to enter in columns headed "Samp.")

	**Description of groups or individuals** N = sampling frame	**Allocation**				**Observation**				**Number Analyzed**
		**Intervention**		**Comparison**		**Intervention**		**Comparison**		
		**n**	**Samp**	**n**	**Samp**	**n**	**Samp**	**n**	**Samp**	
**Groups 1**										
**2**										
**3**										
**Individuals**										

For designs using follow-up of the study population, calculate the completion rate(s) for the study population:

$$\frac{\text{Number analyzed}}{\text{Number allocated}} \times 100$$

Part II. Descriptive Information

12. How did the investigators assess whether exposure to the intervention actually occurred? See instructions for Part 11/Question 8 for additional examples of terms. Provide the definition of the exposure variable(s) as described by the authors and the level of exposure to the intervention. If exposure was different in different subgroups, report the exposure for each group separately. Check all that apply.

Example: *Exposure of mothers to a prenatal or postpartum intervention was assessed by resource utilization: 20% of mothers in the community attend prenatal classes at a clinic; 95% of mothers receive a postpartum home visit.*

13. Provide all of the requested demographic and risk factor information for the intervention and comparison segments of the study population; **baseline data are preferred.** Provide page/table numbers for this question. Provide information for the study population as a whole **only** if the authors do not report the data for the intervention and comparison groups separately. In this situation, calculate the proportions for the intervention and comparison populations if sufficient information is provided by the authors. If the authors provide only descriptive information about the reference population (i.e., the population from which the study population was drawn) instead of the study population, provide that data (see third example, below). If the authors report demographic and risk factor information for more than four groups, duplicate this page for additional space. For each variable, provide the p value or confidence interval for the difference between groups if available in the last column (enter "NS" if not significant, "NR" if not reported, or "NA" if not applicable).

At the top of each column, describe the group for which you are providing the demographic information.

Examples:

- *The authors implemented an intervention in one school (n = 300 students) and used a second school as a comparison (n = 295); they provided separate demographic information for the intervention and comparison schools. Enter "Intervention school" and "Comparison school" at the tops of the two columns and fill in the appropriate data.*
- *The authors implemented an intervention in one school (n = 300 students) and used a second school as a comparison (n = 295), but only provided demographic information for the two groups as a whole (n = 595). Enter "Entire study population, n = 595" at the top of the column.*
- *The authors implemented an intervention in one school (n = 300 students) and used a second school as a comparison (n = 295), but did not provide demographic information for the students participating in the study. Instead, the authors describe the demographics of the community in which the schools are located. Enter "Reference population only" and any descriptive information about the community for which data are provided.*

Age:	Provide median/range, mean/standard deviation, other measure of central tendency or "not reported." If categories are used, provide the categories and the percent of the study population in each category. If a proxy for age such as school grade is presented, indicate the range and the units.
Sex:	Provide the percent male, female, and/or unknown; or "not reported"
Race/Ethnicity:	Provide the percentage for each race/ethnic group or check "not reported" if the authors do not provide this information. If information is provided for part of the population, but not reported for some proportion, check all that apply including the "Other/Unknown" category and specify the proportion unknown. If information is unknown, circle "Unknown"; if the response is other, circle "Other" and specify.
Socioeconomic status:	Check "low," or "middle/upper," as **reported by the authors** or "not reported." Use reasonable judgment to select a category if the authors provide ancillary information (e.g., educational attainment).
Other:	Provide any other demographic or risk factor information reported by the authors.

Examples: *migrant status, educational attainment, occupation, risk behavior categories (e.g., men who have sex with men, drivers with criminal convictions for alcohol-impaired driving), and other potential confounding factors.*

Part II. Descriptive Information

12. Assessment of exposure to the intervention. Provide the definition of each exposure variable and the level of exposure in the space provided for each.

- ☐ Resource utilization *Describe:*
- ☐ Observation *Describe:*
- ☐ Interview *Describe:*
- ☐ Self-administered questionnaire *Describe:*
- ☐ Laboratory test *Describe:*
- ☐ Record review *Describe:*
- ☐ Other *Describe:*
- ☐ Not reported/Did not assess

13. Study population demographics:

	Group	Group	Group	Group	P value or CI
Age	______ ______ ______ ☐ Not reported	______ ______ ______ ☐ Not reported	______ ______ ______ ☐ Not reported	______ ______ ______ ☐ Not reported	
Sex	____ % male ____ % female ____ % unknown ☐ Not reported	____ % male ____ % female ____ % unknown ☐ Not reported	____ % male ____ % female ____ % unknown ☐ Not reported	____ % male ____ % female ____ % unknown ☐ Not reported	
Race (%)	☐ Not reported	☐ Not reported	☐ Not reported	☐ Not reported	
☐ American Indian or Alaska Native	______ %	______ %	______ %	______ %	
☐ Asian	______ %	______ %	______ %	______ %	
☐ Black or African American	______ %	______ %	______ %	______ %	
☐ Native Hawaiian or Other Pacific Islander	______ %	______ %	______ %	______ %	
☐ White	______ %	______ %	______ %	______ %	
☐ Other/Unknown *Specify:*	________ ______ %	________ ______ %	________ ______ %	________ ______ %	
Ethnicity (%)					
☐ Hispanic or Latino	______ %	______ %	______ %	______ %	
☐ Not Hispanic or Latino	______ %	______ %	______ %	______ %	
☐ Other/Unknown *Specify:*	________ ______ %	________ ______ %	________ ______ %	________ ______ %	
Socioeconomic status	☐ Low ☐ Middle/upper ☐ Not reported	☐ Low ☐ Middle/upper ☐ Not reported	☐ Low ☐ Middle/upper ☐ Not reported	☐ Low ☐ Middle/upper ☐ Not reported	
Other population demographic and risk factor characteristics *Specify:*					

Part II. Descriptive Information

14. Some interventions are directed at a specific study population, but ultimately affect health or other related outcomes (e.g., behaviors) that are measured in a different population. For example, a provider education intervention is directed at health care providers (the "study population"), but the health outcome occurs in their patients (the "ultimately affected" population). Another example is when an educational intervention directed at parents (the "study population") ultimately affects their children (the "ultimately affected" population). **Does this study report demographic information for or measure an outcome in a population of persons who were ultimately affected by the intervention applied to the study population?** If no, skip to question 18.

Examples:

- *A professional education intervention about the indications and contraindications for childhood immunizations was administered to half of the physicians in a group practice (the other half served as controls). The researchers measured vaccine coverage rates in the children served by the practice. The researchers presented demographic information for the physicians (i.e., the study population) AND for the children (i.e., the ultimately affected population). Report the demographic information for the physicians in question 13 and for the children in questions 14-17.*
- *If the intervention was implemented in and the effects measured in the same group of people, the answer to this question is "no”*

15. How many groups were in the "ultimately affected" population?

16. Indicate the number of members in each of the "ultimately affected" population groups, and describe those members.

17. Provide all of the requested demographic information for the "ultimately affected" population. See instructions for Part II/Question 13 for details.

Part II. Descriptive Information

14. "Ultimately affected" population described or outcomes reported?
☐ Yes (Go to question 15) ☐ No (Go to question 18)

15. Number of groups in the "ultimately affected" population?

16. Number and description of members in each group:

17. "Ultimately affected" population demographics:

	Group	Group	P value or CI
Age	________<br>________<br>________<br>☐ Not reported	________<br>________<br>________<br>☐ Not reported	
Sex	____ % male<br>____ % female<br>____ % unknown<br>☐ Not reported	____ % male<br>____ % female<br>____ % unknown<br>☐ Not reported	
Race (%)	☐ Not reported	☐ Not reported	
☐ American Indian or Alaska Native	______ %	______ %	
☐ Asian	______ %	______ %	
☐ Black or African American	______ %	______ %	
☐ Native Hawaiian or Other Pacific Islander	______ %	______ %	
☐ White	______ %	______ %	
☐ Other/Unknown<br>*Specify:*	_________<br>______ %	_________<br>______ %	
Ethnicity (%)			
☐ Hispanic or Latino	______ %	______ %	
☐ Not Hispanic or Latino	______ %	______ %	
☐ Other/Unknown<br>*Specify:*	_________<br>______ %	_________<br>______ %	
Socioeconomic status	☐ Low<br>☐ Middle/upper<br>☐ Not reported	☐ Low<br>☐ Middle/upper<br>☐ Not reported	
Other population demographic and risk factor characteristics<br>*Specify:*	________<br>________<br>________<br>________<br>________<br>________<br>________	________<br>________<br>________<br>________<br>________<br>________<br>________	

INSTRUCTIONS

Part II. Descriptive Information

C. Results

18. Primary study results: From Part I/Question 3 of this form, describe each of the primary outcome measures used in this study and the effect measure as reported by the author. Indicate the table number (in the paper) from which the data are taken, if applicable. For each outcome measure, report the results for each arm of the intervention group (as applicable) and for each of the comparison groups (as applicable); report the results for each time period measured as applicable to the study design (i.e., before and after the intervention, only after the intervention, for each time period in a time series design). Fill in the time periods as shown.

Outcome Measure (List from Part I/Question 3and describe effect measure in numbered row)	Effect size reported by authors						Software used, hypothesis testing, p values, CI, etc.
	Studies with pre – post measurements		Studies with multiple measurements over time				
	Pre<br>**Oct 77**	Post<br>**Oct 78**	Time 1<br>**Mar 80**	Time 2<br>**Apr 80**	Time 3<br>**May 80**	Time 4<br>**Jun 80**	
1 a. Prevalence rates of self-reported child restraint device use (baseline rate is pre-intervention rate [Oct '77] for comparison group). Table 3, page 22 in the paper.							
Intervention Arm 1<br>Intervention Arm 2<br>Intervention Arm 3	50%<br>45%	75%<br>68%					Intervention arm 1 versus comparison: x2 =xx,p =xx
Comparison Group 1<br>Comparison Group 2<br>Comparison Group 3	50%	50%					Intervention arm 2 versus comparison: x2 =xx,p =xx
lb. Validation of la using observed rates of child restraint device use (baseline rate is pre- intervention rate {Oct '77] for comparison group). Table 2, page 22 in the paper.							
Intervention Arm 1<br>Intervention Arm 2<br>Intervention Arm 3	49%<br>49%	70%<br>62%					Intervention arm 1 versus comparison: x2 =xx,p =xx
Comparison Group 1<br>Comparison Group 2<br>Comparison Group 3	45%	53%					Intervention arm 2 versus comparison: x2 =xx,p =xx
2. Vaccine coverage rates for children over time (March '80 =baseline rate, intervention applied before time 2; no comparison group). Table 1, page 1543 in the paper.							
Intervention Arm 1<br>Intervention Arm 2<br>Intervention Arm 3			44%	46%	76%	56%	SAS (Proc freq) Change in intervention group time 4 versus time 1 (baseline): x2=xx,p =xx
Comparison Group 1<br>Comparison Group 2<br>Comparison Group 3							
3. Percent of students self-reporting drinking and driving. Table 3, page 29 in paper.							
Intervention Arm 1<br>Intervention Arm 2<br>Intervention Arm 3	NA<br>NA	13%<br>16%					EpiInfo Intervention arm 1 versus comparison: x2=xx,p=xx

Comparison Group 1<br>Comparison Group 2<br>Comparison Group 3	NA	29%					EpiInfo Intervention arm 2 versus comparison: x2=xx,p=xx

19. Did the authors conduct a power analysis, discuss other statistical procedures, or cite other literature to determine the appropriate sample size PRIOR to implementation of the intervention? If no, IN YOUR OPINION, was the sample size sufficient to find the desired effect? Provide a brief justification of this determination.

Example: *Study included interviews with 98% of women with live births in 2 communities and conducted follow-up with 80% of the original study population. Sample sufficient to find a relatively small effect.*

Part II. Descriptive Information

DATA SHEET

C. Results

18. Primary study results

Outcome Measure (List from Part I/Question 10 and describe effect measure in numbered row)	Effect size reported by authors						Software used, hypothesis testing, p values, CI, etc.
	Studies with pre – post measurements		Studies with multiple measurements over time				
	Pre	Post	Time 1	Time 2	Time 3	Time 4	
1.							
Intervention Arm 1<br>Intervention Arm 2<br>Intervention Arm 3							Intervention arm 1 versus comparison: x2 =xx,p =xx
Comparison Group 1<br>Comparison Group 2<br>Comparison Group 3							Intervention arm 2 versus comparison: x2 =xx,p =xx
2							
Intervention Arm 1<br>Intervention Arm 2<br>Intervention Arm 3							Intervention arm 1 versus comparison: x2 =xx,p =xx
Comparison Group 1<br>Comparison Group 2<br>Comparison Group 3							Intervention arm 2 versus comparison: x2 =xx,p =xx
3							
Intervention Arm 1<br>Intervention Arm 2<br>Intervention Arm 3							SAS (Proc freq) Change in intervention group time 4 versus time 1 (baseline): x2=xx,p =xx
Comparison Group 1<br>Comparison Group 2<br>Comparison Group 3							
4							
Intervention Arm 1<br>Intervention Arm 2<br>Intervention Arm 3							EpiInfo Intervention arm 1 versus comparison: x2=xx,p=xx
Comparison Group 1<br>Comparison Group 2<br>Comparison Group 3							EpiInfo Intervention arm 2 versus comparison: x2=xx,p=xx

19. Power calculation, other statistical analysis, or citation?

[ ] Yes

[ ] No; was sample size sufficient? (Justification):

20. Were secondary results of interest reported (including subpopulation differences, dose-response relationships, or others)? If yes, describe those results. Include page and table numbers.

Examples:

- *The effect was stronger among African-American children (the postcard reminder resulted in 70% of children being up to date on immunizations at age 2 years compared to 20% of children who received "usual care. ")*
- *The intervention had less effect among white children where 40% of children who did and did not receive the intervention were up to date on immunizations at age 2 years).*

D. Feasibility and Other Key Issues Addressed in the Paper

21. Which of the following feasibility and other key issues were addressed in the paper? To flag issues that might be of importance in describing the intervention or its implementation, check off any of the following issues that are described by the authors. This will assist the chapter development team in quickly identifying papers that address these issues. Check all that apply. **Include the page numbers where this information can be found in the paper.**

- Costs of the intervention (include monetary, nonmonetary or human resources)
- Potential harms of the intervention (includes health and social consequences)
- Other benefits
- Implementation of the intervention
- Barriers to implementation
- Community acceptance or involvement in development or implementation of the intervention
- Formation or use of existing coalitions to develop, implement, or evaluate interventions
- Ethical constraints
- Other
- Not discussed (i.e., no other data were presented)

22. In the space provided, include any other information that you feel we should be aware of or that will aid you in evaluating the quality of the intervention in the next section of this form. ***Example:*** *Some evaluations may be able to measure how the intervention was monitored (e.g., fidelity, quality assurance). Describe such efforts here.*

23. Identify references from the reference list that might be related to the particular intervention, subtopic, or topic that is the focus of this review. Provide this information by circling or highlighting the relevant references directly on a photocopy of the references pages from the paper and returning it with this form or by listing the reference numbers (or the author and year) from the reference list in the space provided.

Example: *During a review about the effectiveness of patient reminders in improving vaccine coverage, a reference about patient reminders to improve measles vaccine coverage would be directly relevant, but references about efficacy of vaccine or effectiveness of community education in improving vaccine coverage, or about burden of measles disease in the U.S. would not be directly relevant.*

Part II. Descriptive Information

20. Secondary results ☐ Yes ☐ Not reported
If Yes, Specify:

E. Feasibility and Other Key Issues Addressed in the Paper

21. ☐ Costs

☐ Potential harms

☐ Other benefits

☐ Implementation

☐ Barriers to implementation

☐ Community acceptance or involvement

☐ Formation or use of existing coalitions to develop, implement, or evaluate interventions

☐ Ethical constraints

☐ Other *Describe:*

☐ Not discussed

22. Other important information:

23. Relevant references:

Part III: Study Quality Instructions

Study quality is evaluated using six categories of common problems (Descriptions, Sampling, Measurement, Analysis, Interpretation of Results, and Other). Study validity poses a complex problem when evaluating the quality of studies. It is possible that elements of each of the six categories contribute to problems with study validity. Therefore, we have tried to elicit information in each category that may contribute to poor study validity which potentially limit our ability to interpret the results of the study.

Some problems with a study can be included under several of the categories. Use your best judgment to list the problem under the **MOST** appropriate category.

Example: Students at schools that had an intensive educational program to reduce drug use could have been less likely than other students to report drug use (independent of actual use). This problem could be marked as a limitation of this study under the category "Measurement" because of problems with the validity and reliability of self-reported outcomes. Alternatively, this problem could be marked as a limitation of the study under the category "Interpretation of Results" because of poor randomization, other activities ongoing in the schools, uncontrolled differences in the intervention and comparison populations prior to implementation of the intervention, etc. **The reviewer must decide if one or both of these categories are limited based on the information provided in the paper. If questions arise, err on the side of providing more information and checking the maximum number of categories.**

The relative merits of different study designs will be considered separately from the quality of execution of the study. **Thus, given that the study you reviewed has a particular study design (Part 1/Question 1), answer these questions based on the quality of execution of this study's design, NOT whether this was the best possible study design that could have been used.**

One or more questions are posed for each category. Each question is designed to elicit information about potential limitations in the quality of a study. In the column to the right of each question, the numbers corresponding to items in Part II of this form relevant to answering that question are provided. Answers that suggest quality limitations are labeled "limitation." **Potential quality limitations for a question should be noted if they are of sufficient magnitude to diminish your confidence in the results.**

Briefly explain each of your assessments in the space provided; **always provide comments for limitations of a question.** If possible, the impact of the limitation on the results should be estimated. (e.g., a study in which many members of the control group received an intervention that was similar to that offered to the intervention group would probably underestimate any reported effect of the intervention).

Note: When it appears as a response option, N/A=Not Applicable.

EXPLAIN ALL ASSESSMENTS!

**1.**	**Descriptions**	**Related Questions**
A.	Was the study population (i.e., the intervention and comparison population) well described?<br>The study population should be described by time (e.g., when the study population received the intervention), place, and person. Information about "person" should include at least age (for all studies) and should include other relevant characteristics of participants that are key to a particular study *(e.g., SES, gender, other).* Important potential confounding factors should also be described.	II/1, 5, 6, 11a/b, 13
B.	Was the intervention well described? The intervention should be described in terms of what was done, how it was delivered, who was targeted, and where it was done.	II/1, 2, 3, 4

**2.**	**Sampling**	**Related Questions**
A.	Did the authors specify (i.e., describe characteristics and size of) the sampling frame or universe of selection for the study population?	II/1, 11b
B.	Did the authors specify the screening criteria for study eligibility (if applicable)?	II/1, 11a
C.	Was the population that served as the unit of analysis the entire eligible population or a probability sample at the point of observation?<br><br>**To answer this question follow these steps:**<br>1. Using question 11 b in Part II (page 4) refer to the column "Number analyzed" to identify the unit(s) of analysis(es).<br><br>2. The question refers to the sampling method ("Samp.") under the column labeled "Observation" for that unit of analysis. If the sampling method is "E" or "P" the answer to this question is "Yes;" otherwise, the answer to this question is "No."	II/11b
D.	Are there other selection bias issues not identified above? This might include a very low participation rate (or a high refusal rate), an all-volunteer sample (as opposed to a convenience sample selected by the investigators), an inappropriate control or comparison group, or extremely restricted sampling inappropriate for measuring the effectiveness of the intervention being studied.	II/11 a/b

Part III. Study Quality

DATA SHEET

EXPLAIN ALL ASSESSMENTS!

**1.**	**Descriptions**	**Yes**	**No**	**Related Questions**
A.	Was the study population well described?	☐	☐	II/1, 5, 6, 11a/b, 13
B.	Was the intervention well described (what, how, who, where)?	☐	☐	II/1, 2, 3, 4

Explain:

**2.**	**Sampling**	**Yes**	**No**	**N/A**	**Related Questions**
A.	Did the authors specify the sampling frame or universe of selection for the study population?	☐	☐	☐	II/1, 11b
B.	Did the authors specify the screening criteria for study eligibility?	☐	☐	☐	II/1, 11a
C.	Was the population that served as the unit of analysis the entire eligible population or a probability sample at the point of observation?	☐	☐	☐	II/11b
D.	Are there other selection bias issues not otherwise addressed? **Describe.**	☐	☐	☐	II/11a/b

Explain:

DID YOU PROVIDE COMMENTS FOR ALL ASSESSMENTS?

3.	Measurement	Related Questions
A.	Was there an attempt to measure exposure to the intervention? **(If NA or No, go to 3C)**	II/12
B.	Were the exposure variables valid measures of the intervention under study?<br>**The authors should have reported one or more of the following:**<br>• Clear definition of the exposure variable.<br>• Measurement of exposure in different ways. ***Example:*** *consistency checks for self-reports; use of corroborating respondents; program or organizational record searches compared to self-reports.*<br>• Citations or discussion as to why the use of these measures is valid. ***Example:*** *the authors considered evidence from similar studies, or available standards of measurement.*<br>• Other	II/12
	Were the exposure variables reliable (consistent and reproducible) measures of the intervention under study? **The authors should have reported one or more of the following:**<br>• Measures of internal consistency. ***Example:*** *Cronbach* 's *alpha; confirmatory factor analysis.*<br>• Measurement of exposure in different ways. ***Example:*** *see above.*<br>• Inter-rater reliability checks (if exposure was determined by an observer). ***Example:*** *percent agreement, Kappa*<br>• Citations or discussion as to why the use of these measures is reliable. ***Example:*** *see above*<br>• Other	II/12
C.	Were the outcome and other independent (or predictor) variables valid measures of the outcome of interest?<br>**The authors should have reported one or more of the following:**<br>• Clear definition of the outcome variable.<br>• Measurement of the outcome in different ways. ***Example:*** *Correlational analysis between measured outcomes to demonstrate convergent (i.e., 2 or more measures reflect the same underlying process) or divergent validity (i.e., 2 or more measures reflect different dimensions). An example of the former is that 5 items on self-efficacy correlate highly with each other; an example of the latter is that self-efficacy measures do not correlate highly with attitude measures.*<br>• Citations or discussion as to why the use of these measures is valid. ***Example:*** *see above*<br>• Other. ***Example:*** *If authors fail to blind observers/interviewers to treatment vs. comparison group, when applicable, the answer to this question should be "no.* "	I/10
	Were the outcome and other independent (or predictor) variables reliable (consistent and reproducible) measures of the outcome of interest?<br>**The authors should have reported one or more of the following:**<br>• Measures of internal consistency. ***Example:*** *see 3B*<br>• Measurement of the outcome in different ways. ***Example:*** *see 3B and 3C (above).*<br>• Considered consistency of coding, scoring or categorization between observers (e.g., interrater reliability checks) or between different outcome measures. ***Example:*** *percent agreement, Kappa*<br>• Considered how setting and sampling of study population might affect reliability.<br>• Citations or discussion as to why the use of these measures is reliable. ***Example:*** *see 3B.*<br>• Other	II/8, 9 10, 18,20

4.	Data Analysis	Related Questions
A.	Check "yes," "no," or "not applicable" for each of the following:<br>Did the authors conduct appropriate analysis by:	
	• Conducting statistical testing (when appropriate)?	II/18, 20
	• Reporting which statistical tests were used?	II/18, 20
	• Controlling for design effects in the statistical model?<br>***Examples:*** ·<br>*1. The study population was sampled using complex stratified sampling, however, the authors did not control for the sampling method in the analysis.*<br>*2. The answer should be "no" if the study had a matched design but an unmatched analysis.*	II/18, 20
	• Controlling for repeated measures in the analysis, for study designs in which the same population was followed with repeated measurements over time?	II/18, 20
	• Accounting for different levels of exposure in segments of the study population in the analysis?	II/12
	• If the authors analyzed group-level and individual-level covariates in the same statistical model, was the model designed to handle multi-level data?	II/1, 11b
B.	Were there other problems with data analysis that limit interpretation of the results of the study?<br>Specify	II/18, 20

Part III. Study Quality

DATA SHEET

**3.**	**Measurement**	**Yes**	**No**	**N/A**	**Related Questions**
A.	Did the authors attempt to measure exposure to the intervention?	☐ go to 3B	☐ go to 3C	☐ go to 3C	II/12
B.	Was the exposure variable:				
	• Valid?	☐	☐	☐	II/12
	• Reliable (consistent and reproducible)?	☐	☐	☐	II/12
C.	Were the outcome and other independent (or predictor) variables:				
	• Valid?	☐	☐	☐	I/10
	• Reliable (consistent and reproducible)?	☐	☐	☐	II/8, 9, 10, 18, 20

Explain:

**4.**	**Data Analysis**	**Yes**	**No**	**N/A**	**Related Questions**
A.	Did the authors conduct appropriate statistical testing by:				
	• Conducting statistical testing (when appropriate)?	☐	☐	☐	II/18, 20
	• Reporting which statistical tests were used?	☐	☐	☐	II/18, 20
	• Controlling for design effects in the statistical model?	☐	☐	☐	II/18, 20
	• Controlling for repeated measures in populations that were followed over time?	☐	☐	☐	II/18, 20
	• Controlling for differential exposure to the intervention?	☐	☐	☐	II/12
	• Using a model designed to handle multi-level data when they included group-level and individual covariates in the model?	☐	☐	☐	II/1, 11b
B.	Are there other problems with the data analysis? **Describe.**	☐	☐	☐	II/18, 20

Explain:

5.	Interpretation of Results	Related Questions
A.	Did at least 80% of enrolled participants (i.e., intervention AND comparison groups) complete the study? This may be reported as a "lost-to-follow-up" or "drop-out" rate. If the authors did not report ≥ 80% follow-up but conducted an alternative analysis that concluded that the high attrition did not influence the results of the study, check "yes."<br><br>For many study designs, this criterion is not applicable (i.e., time series, before-after designs with or without a concurrent comparison group, surveys); for these studies, check the response option "Not Applicable."	II/11 a/b, 18, 20
B.	Confounding:	
	• Did the authors assess whether the units of analyses were comparable prior to exposure to the intervention? For example, they should have assessed like!>' confounding via report of p values and confidence intervals for the descriptive variables of age and sex or other key individual/community characteristics.	II/13
	• Considering the study design, were appropriate methods for controlling confounding variables and limiting potential biases used? Confounding can be addressed by appropriate use of randomization, restriction, matching, stratification, or multivariable methods. Sometimes use of a single method may be inadequate. Some biases can be limited by institution of data collection or study procedures that support validity of the study (e.g. training and/or blinding of interviewers or observers, interviewers and observers are different from intervention implementors, etc.)<br>***Example:*** *If between-group differences persist after randomization or matching, statistical control should also have been used*	I/8:<br>II/11b, 18, 20<br>III/6A
C.	Biases:<br><br>Did the authors identify and discuss potential biases or unmeasured/contextual confounders that may account for or influence the observed results and explicitly state how they assessed these potential confounders and biases?*<br>Please describe these factors and, if possible, comment on the likely direction of bias. If there are additional biases NOT COVERED IN OTHER CATEGORIES that the authors did not address, please list these as well.<br>***Examples:***<br>*1. A time series study of an intervention intended to enhance immunization delivery during a period of considerable attention to immunizations could incorrectly attribute increases in vaccine coverage to the intervention under study and thus overestimate the effect of the intervention.*<br>*2. A study of an educational program to improve levels of physical activity during a period when the control group was also likely to receive considerable education about physical activity could under-estimate the effectiveness of the program.*	

6. Other
Are there other issues that limit your ability to interpret the results of the study that were not identified handled in one of the other categories? **Please limit your comments** in this box to those limitations of the study that cannot be evaluated in other categories, and for which you can make a detailed justification. If you have a concern but are not able to clearly state why it should be a limitation of the study, contact the staff scientist to discuss the issue.

* Many excellent epidemiology and evaluation texts describe biases inherent in different study designs. For a concise list and definitions of various biases, refer to: A dictionary of epidemiology. 2nd Edition. Last JM, ed. New York, New York: Oxford University Press, 1988.

Part III. Study Quality

DATA SHEET

5.	Interpretation of Results	Yes	No	N/A	Cross Reference
A.	Did at least 80% of enrolled participants complete the study?	☐	☐	☐	II/11a/b, 18, 20
B.	Did the authors assess:				
	• Whether the units of analyses were comparable prior to exposure to the intervention?	☐	☐	☐	II/13
	• Correct for controllable variables or institute study procedures to limit bias appropriately (e.g., randomization, restriction, matching, stratification, or statistical adjustment)?	☐	☐	☐	I/8; II/11b, 18, 20; III/6A

Explain:

C. Check "yes" and describe all potential biases or unmeasured/contextual confounders described by the authors. You may also check "no" and describe other potential biases or unmeasured/contextual confounders NOT identified by the authors. For all responses, indicated the likely direction of effect on the results, if possible.

Yes ☐ **No** ☐

authors:

reviewers:

6. Other

Other important limitations of the study **not** identified elsewhere (specify):

DID YOU PROVIDE COMMENTS FOR ALL ASSESSMENTS?

Study Design Algorithm

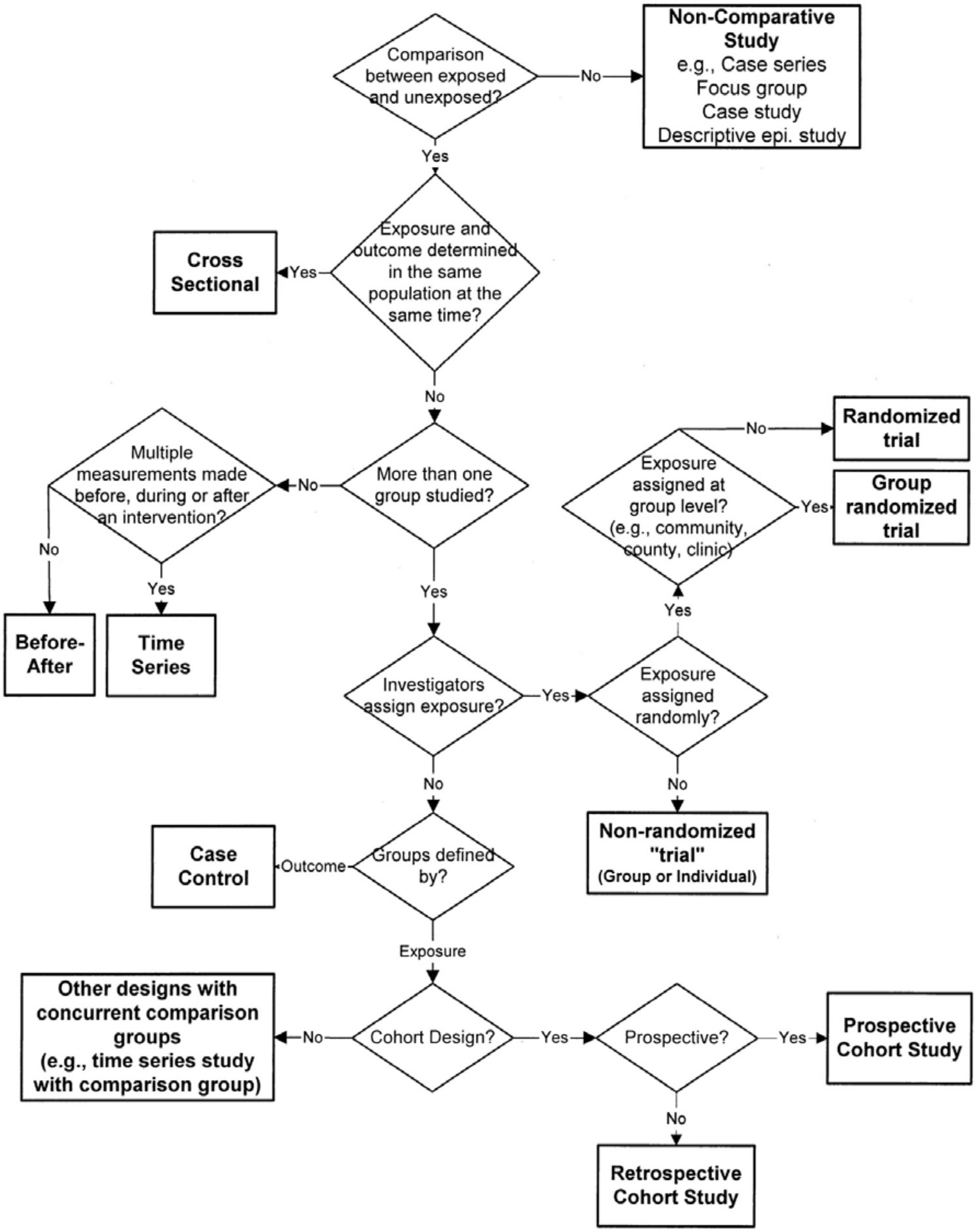

Supplement: Supplementary file 1 [file DataSheet_1.pdf]
