# Supplementary material for: Antipsychotics for Amphetamine Psychosis. A Systematic Review
Source: Front Psychiatry. 2019 Oct 15;10:740. doi: 10.3389/fpsyt.2019.00740 (PMC6804571; doi:10.3389/fpsyt.2019.00740)
Supplement: Supplementary file 2 [file Table_1.docx]

|  |  | **Risk of bias assessments for selected studies** | | | | | |  |
| --- | --- | --- | --- | --- | --- | --- | --- | --- |
|  |  | **Selection** | **Performance** | **Detection** | **Attrition** | **Reporting** | **Others** |  |
| **Farnia et al. 2014** | | **(-)** | **(-)** | **(-)** | **(-)** | **(-)** | **(-+)** |  |
| **Leelahanaj et al 2005** | | **(-)** | **(-)** | **(-)** | **(-)** | **(-)** | **(-+)** |  |
| **Samiei et al. 2016** | | **(-)** | **(+)** | **(+)** | **(-)** | **(-)** | **(-+)** |  |
| **Sulaiman et al. 2013** | | **(-)** | **(-)** | **(-)** | **(-)** | **(-)** | **(-+)** |  |
| **Verachai et al. 2014** | | **(-)** | **(-)** | **(-)** | **(-)** | **(-)** | **(-+)** |  |
| **Wang et al. 2016** | | **(-)** | **(+)** | **(+)** | **(-)** | **(-)** | **(-+)** |  |
|  |  |  |  |  |  |  |  |  |
| **Key** |  | **Performance:blinding particpants and personnel** | | | |  |  |  |
| **(+) High risk of bias** |  | **Dectection: blinding of outcome assessors** | | | |  |  |  |
| **(-) Low risk of bias** |  | **Attrition: missing data (participants, demographic, sex, age etc...)** | | | | | |  |
| **(-+) Unclear risk of bias** |  | **Selection: describe a random component** | | | |  |  |  |
|  |  | **Reporting: primary and secondary outcomes** | | | |  |  |  |
|  |  | **Others: conflict of interest......** | | |  |  |  |  |
|  |  |  |  |  |  |  |  |  |
